# Nervous system development in lecithotrophic larval and juvenile stages of the annelid *Capitella teleta*

**DOI:** 10.1186/s12983-015-0108-y

**Published:** 2015-07-11

**Authors:** Néva P Meyer, Allan Carrillo-Baltodano, Richard E Moore, Elaine C Seaver

**Affiliations:** Biology Department, Clark University, 950 Main Street, Worcester, MA 01610 USA; Department of Molecular Biosciences and Bioengineering, University of Hawaii at Manoa, 1955 East–west Road, Honolulu, HI 96822 USA; Whitney Laboratory for Marine Bioscience, University of Florida, 9505 Ocean Shore Boulevard, Saint Augustine, FL 32080 USA

## Abstract

**Background:**

Reconstructing the evolutionary history of nervous systems requires an understanding of their architecture and development across diverse taxa. The spiralians encompass diverse body plans and organ systems, and within the spiralians, annelids exhibit a variety of morphologies, life histories, feeding modes and associated nervous systems, making them an ideal group for studying evolution of nervous systems.

**Results:**

We describe nervous system development in the annelid *Capitella teleta* (Blake JA, Grassle JP, Eckelbarger KJ. *Capitella teleta*, a new species designation for the opportunistic and experimental *Capitella* sp. I, with a review of the literature for confirmed records. Zoosymposia. 2009;2:25–53) using whole-mount in situ hybridization for a *synaptotagmin 1* homolog, nuclear stains, and cross-reactive antibodies against acetylated α-tubulin, 5-HT and FMRFamide. *Capitella teleta* is member of the Sedentaria (Struck TH, Paul C, Hill N, Hartmann S, Hosel C, Kube M, et al. Phylogenomic analyses unravel annelid evolution. Nature. 2011;471:95–8) and has an indirectly-developing, lecithotrophic larva. The nervous system of *C. teleta* shares many features with other annelids, including a brain and a ladder-like ventral nerve cord with five connectives, reiterated commissures, and pairs of peripheral nerves. Development of the nervous system begins with the first neurons differentiating in the brain, and follows a temporal order from central to peripheral and from anterior to posterior. Similar to other annelids, neurons with serotonin-like-immunoreactivity (5HT-LIR) and FMRFamide-like-immunoreactivity (FMRF-LIR) are found throughout the brain and ventral nerve cord. A small number of larval-specific neurons and neurites are present, but are visible only after the central nervous system begins to form. These larval neurons are not visible after metamorphosis while the rest of the nervous system is largely unchanged in juveniles.

**Conclusions:**

Most of the nervous system that forms during larvogenesis in *C. teleta* persists into the juvenile stage. The first neurons differentiate in the brain, which contrasts with the early formation of peripheral, larval-specific neurons found in some spiralian taxa with planktotrophic larvae. Our study provides a clear indication that certain shared features among annelids - *e.g.*, five connectives in the ventral nerve cord - are only visible during larval stages in particular species, emphasizing the need to include developmental data in ancestral character state reconstructions. The data provided in this paper will serve as an important comparative reference for understanding evolution of nervous systems, and as a framework for future molecular studies of development.

**Electronic supplementary material:**

The online version of this article (doi:10.1186/s12983-015-0108-y) contains supplementary material, which is available to authorized users.

## Background

Nervous systems are critical for many aspects of animal life; they sense and respond to the environment, regulate organ systems, and control movement and behavior. Understanding how nervous systems develop and were elaborated upon during the course of evolution are fundamental questions in animal biology. The architecture of a nervous system often reflects the result of selection on traits such as body plan, lifestyle, and mode of feeding [1]. For example, animals with a sessile adult lifestyle (*e.g.*, filter-feeding annelids) can have reduced brains while closely related motile animals that hunt prey can have more complex brains with larger numbers of neurons and morphologically distinct brain regions [2–5]. Similarly, animals with different larval and adult body plans can exhibit larval nervous systems that are restructured into the adult nervous system at metamorphosis or that degenerate and are replaced by the adult nervous system at metamorphosis [6–8]. The relationships between larval and adult nervous systems within and between species are currently under debate (*e.g.,* [5, 9–13]). Determination of the structure and development of nervous systems in diverse taxa is crucial if we hope to understand the evolutionary history of this vital organ system.

One group of animals, the Spiralia, are particularly useful for studying evolution of body plans and organ systems. Spiralia is a large clade that includes annelids, mollusks, nemerteans, brachiopods, platyhelminthes, bryozoans, phoronids, and entoprocts [14]; see [15, 16] for a review of the use of Spiralia versus Lophotrochozoa. Taxa within this group exhibit a wide range of life histories, larval forms, body plans, and accompanying nervous systems [1, 17, 18]. Development in many spiralians begins with spiral cleavage, a conserved cleavage program in which most blastomeres have a largely invariant lineage. Spiral cleavage facilitates the comparison of fates generated by homologous blastomeres across taxa with very different body plans [16, 19–21]. Modern techniques are increasingly being used to study the molecular mechanisms underlying the evolution of these distinct body plans [22]. For these reasons, studies of spiralian nervous system development can provide insights into the evolution of this organ system.

Within the spiralians, annelids are important for studies of body plan and nervous system evolution for several reasons. Annelida includes ~16,500 described species that inhabit a range of oceanic habitats from intertidal to deep benthic zones as well as freshwater and moist terrestrial habitats [1]. Annelids exhibit a variety of morphologies, life histories, feeding modes, and associated nervous systems. Recent phylogenomic analyses and character-state reconstructions have found several taxa with very diverse traits at the base of Annelida, including Sipuncula, Amphinomidae, Chaetopteridae, Magelonidae, and Oweniidae [23, 24]. The rest of the annelid taxa are currently subdivided into two groups with different lifestyles, Sedentaria and Errantia. Animals within Sedentaria have more sedentary lifestyles and other traits associated with sessility such as reduced sensory organs and parapodia. Members of Errantia have a more motile lifestyle that involves hunting or scavenging and have well-developed sensory organs and parapodia [23, 24].

Development of a few annelid species such as *Capitella teleta*, *Helobdella* (members of Sedentaria) and *Platynereis dumerilli* (a member of Errantia) [23, 24] have been studied in some detail [25]. A comprehensive knowledge of normal development in these and other species would provide a foundation for comparative and experimental studies both within these annelids and with other spiralians. The focus species for this study, *C. teleta* [26], is a deposit-feeding polychaete that displays indirect development and lacks a trochophore larva. Although these animals are deposit-feeders, they have a well-defined central nervous system with several hundred cells in the mid-stage larval brain and over 1200 cells in the late-stage larval and early juvenile brain.

Several previous studies have examined nervous system development in spiralians using cross-reactive antibodies against neurotransmitters such as serotonin (5-HT) and FMRFamide, which label subsets of neurons, and against different forms of tubulin such as acetylated α-tubulin, which labels neurites (*e.g.,* [8, 13, 27–37]). From these studies, it has been possible to formulate hypotheses concerning the possible ancestral state of nervous system architecture and development within annelids and mollusks. Another marker useful for examining nervous system development are homologs of Synaptotagmin 1 (Syt1), which are important for exocytosis of synaptic vesicles [38] and found in most metazoans [39]. Because *syt1* homologs are expressed in most if not all mature neuronal cell bodies in many animals [40–42], they serve as a useful broad neuronal marker. Examination of *syt1* homolog expression can reveal features of a developing nervous system that may be missed when only examining markers for neurites or for neurotransmitters that label a small subset of neurons.

In this study, we describe nervous system development in the model annelid *Capitella teleta* using whole-mount in situ hybridization for a *synaptotagmin 1* homolog, nuclear stains and cross-reactive antibodies against acetylated α-tubulin, 5-HT and FMRFamide. Use of antibodies against 5-HT and FMRFamide allowed us to visualize subsets of neurons, and since these two cross-reactive antibodies are widely used, they are useful for making comparisons across taxa. The anti-acetylated-α-tubulin antibody allowed us to visualize the overall architecture of neurites while the *synaptotagmin 1* in situ allowed us to visualize many if not all neuronal cell bodies, giving a more complete view of nervous system development from gastrulation through metamorphosis.

## Results

### Overview of *C. teleta* development

**Fig. 1:**
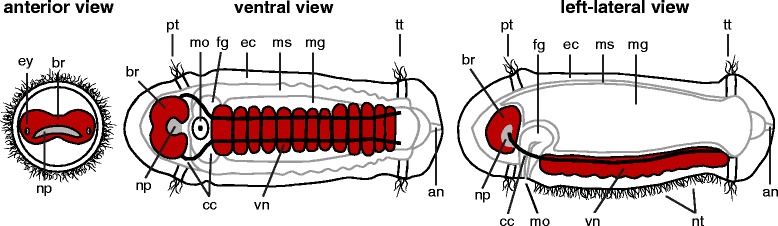
Diagram of a stage 6 *C. teleta* larva. The body plan of a stage 6 larva is shown from an anterior, ventral, and left-lateral view. The central nervous system is in red. an, anus; br, brain; cc, circumesophageal connective; ec, ectoderm; ey, eye; fg, foregut; mg, midgut; mo, mouth; ms, mesoderm; np, neuropil; nt, neurotroch; pt, prototroch; tt, telotroch; vn, ventral nerve cord

A standard embryonic and larval staging system based on morphological features has previously been described for *C. teleta* [43, 44]. Each day of development at 19 °C coincides with a different stage. Stages 1 and 2 include fertilization and cleavage, stage 3 includes gastrulation and stages 4 – 9 encompass larval development. *Capitella teleta* has a non-feeding, swimming larva, which is diagrammed in Fig. 1 for stage 6. *Capitella teleta* larvae are characterized by two ciliary bands, an anterior prototroch (pt) and a posterior telotroch (tt). The prototroch marks the boundary between the head and the trunk, and the telotroch marks the boundary between the trunk and the posterior pygidium. The ventral nerve cord (vn) is positioned between the prototroch and telotroch, and is connected to the anterior brain (br) by a pair of circumesophageal connectives (cc) that surround the mouth (mo). The brain has two lobes with a central neuropil (np) area. Two larval eyes (ey) are positioned immediately anterior of the prototroch, in a lateral position. The first morphological sign of neural development is a thickening of the anterior ectoderm during stage 3 [45]. This thickening is the precursor of the two brain lobes. The mouth also appears during stage 3. Stage 4 is marked by the appearance of the ciliary bands, and the first differentiated neurons become visible during this time. Stage 9 larvae are competent to metamorphose following emergence from a parental brood tube, and juveniles begin feeding the first day after metamorphosis. Progression from a juvenile to a sexually mature adult takes approximately 8 – 10 weeks at 17 °C. During this time, the body plan remains essentially the same, but there is a dramatic increase in body size and number of segments.

### Acetylated-α-tubulin-like immunoreactivity (aTUB-LIR) and serotonin-like-immunoreactivity (5HT-LIR) in early-stage *C. teleta* larvae (stages 4 – 6)

**Fig. 2:**
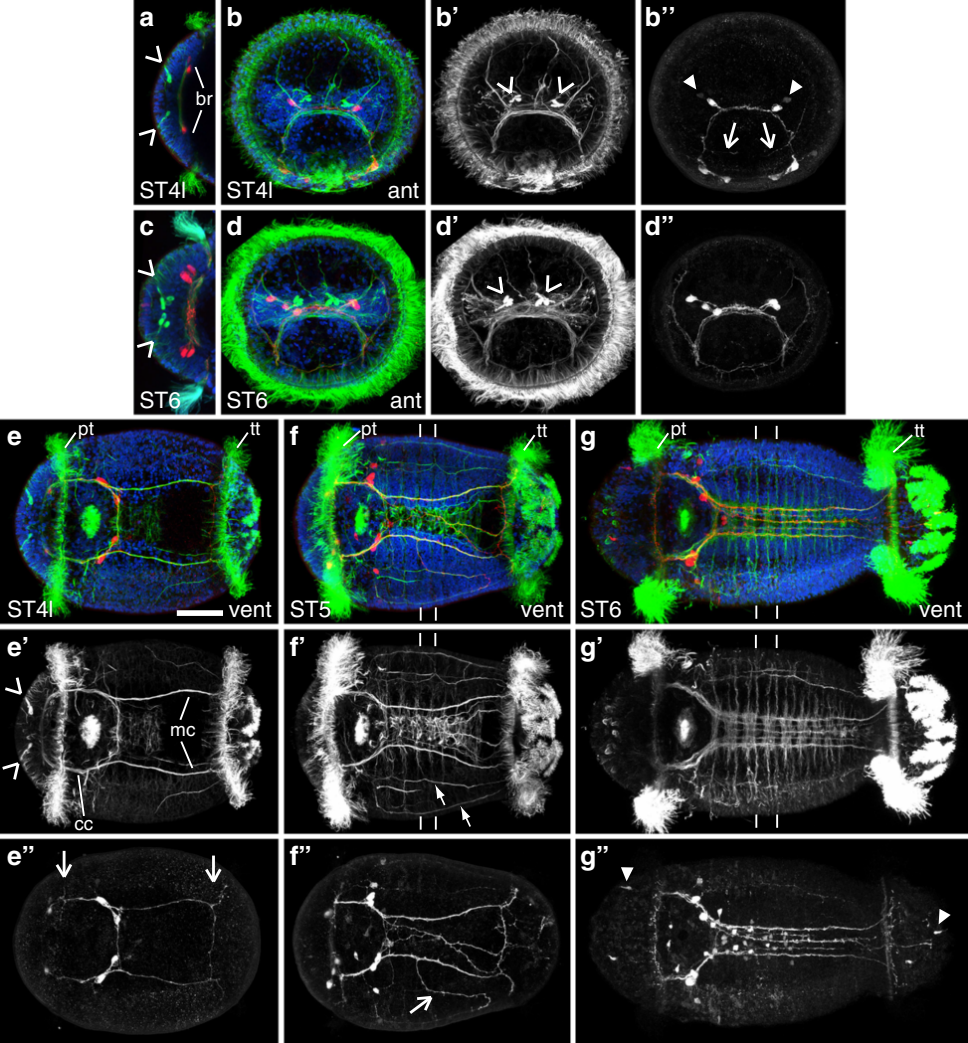
aTUB-LIR and 5HT-LIR in early-stage *C. teleta* larvae (stages 4 – 6). Images are z-stack confocal images of larvae labeled with anti-acetylated-α-tubulin (green), anti-serotonin (red) and nuclear stain (blue). Panels labeled with an apostrophe (*e.g.,* **a'**) are single-channel images of either aTUB-LIR (’) or 5HT-LIR (”) from the merged image without an apostrophe (*e.g.,* **a**) except where otherwise noted. The z-stack in **g”** is of the same animal, but includes more superficial focal planes in order to show cell bodies with 5HT-LIR. Panels **a** and **c** are cropped views of the brain. The two forming brain lobes (br) are indicated in **a**. The scale bar in **e** is 50 μm, and all images are to approximately the same scale. Open arrowheads in **a**, **b’**, **c**, **d’**, **e’** indicate sc^ac+^. In **b”**, closed arrowheads point to a faint pair of neuronal cell bodies with 5HT-LIR and open arrows point to neurites with 5HT-LIR that run along the ventral side of the prototroch. The position of the prototroch (pt) and telotroch (tt) is indicated in **e**, **f** and **g**. The circumesophageal connectives (cc) and main connectives (mc) of the ventral nerve cord are indicated in **e’**. Open arrows in **e”** point to neurites with 5HT-LIR that run along the prototroch and telotroch. In **f**, **f’**, **g** and **g’**, lines mark the posterior boundary of segments three and four. In **f’**, closed arrows point to the right pair of ventral-lateral neurites that run from anterior to posterior. In **f”**, the open arrow indicates an aberrant neurite. Closed arrowheads in **g”** point to peripheral neurons with 5HT-LIR. Stage is indicated in the lower-left corner, and view is indicated in the lower-right corner, except for **a** and **c**, which are dorsal views. Anterior is to the left in all ventral views, and ventral is down in all anterior views. ant, anterior; cc, circumesophageal connectives; mc, main connectives; pt, prototroch; tt, telotroch; vent, ventral

Cross-reactive antibodies against acetylated α-tubulin and the neurotransmitter serotonin allow visualization of neurites and subsets of neurons, respectively. In general, early development of the nervous system progresses from anterior to posterior in *C. teleta*. During stage 4, the basic architecture of the central nervous system, *i.e.,* brain (Fig. 2a br, b), circumesophageal connectives (Fig. 2b, e, e’ cc) and the main connectives of the ventral nerve cord (Fig. 2e, e’ mc), is formed. During stages 5 and 6, more elements of the central nervous system are added, including more neurons with serotonin-like-immunoreactivity (5HT-LIR) and more neurites with acetylated-α-tubulin-like immunoreactivity (aTUB-LIR) in the brain and ventral nerve cord (Fig. 2c, d, f, g). By the end of stage 6, the ventral nerve cord has five connectives and several ganglia and commissures (Fig. 2g). Elements of the peripheral nervous system, including pairs of peripheral nerves in the trunk, are also visible by this stage (Fig. 2g, g').

In *C. teleta*, the first elements of the nervous system that are visible by aTUB-LIR and 5HT-LIR form in the brain during stage 4. Neurites with aTUB-LIR are present in the brain at the beginning of stage 4 (Additional file 1d open arrow), when cilia of the prototroch become visible. The first pair of cells with 5HT-LIR is visible slightly later at mid-stage 4 (Additional file 1a, a” closed arrowhead), when cilia of the telotroch become visible. From mid- to late-stage 4, each of the 5HT-LIR neurons in the brain extends a neurite contralaterally through the brain neuropil (Fig. 2a) and then ventrally along the circumesophageal connectives (Fig. 2b, b”, e, e” and data not shown). By the end of stage 4, faint 5HT-LIR can be seen in a second pair of neurons in the brain (Fig. 2b” closed arrowheads). Also visible by mid- to late-stage 4 in the head are one to two pairs of cells with prominent aTUB-LIR in their soma (Fig. 2a, b’, e’ open arrowheads). These cells form in the region of the developing brain and have been previously described by Amiel *et al.* as “acetylated tubulin-positive sensory cells (sc^ac+^)” [46]. The sc^ac+^ extend processes to the anterior surface of the larva and initially appear to be superficial to cells in the developing brain.

The basic architecture of the central nervous system is formed during stage 4 and can be seen with both aTUB-LIR and 5HT-LIR. At mid-stage 4, neurites with aTUB-LIR make an anterior ring, comprising the rudimentary brain neuropil (np), circumesophageal connectives (cc) and subesophageal commissure (Additional file 2a, a’ sc). Paired neurites with aTUB-LIR also extend along the trunk to the telotroch, forming the rudimentary main connectives (Additional file 2a, a’ mc) of the ventral nerve cord. As stage 4 progresses, the pattern of neurites with aTUB-LIR remains very similar to that seen in mid-stage 4, with the appearance of additional neurites in the trunk (Fig. 2e, e’, Additional file 1b and b’) and paired neurites that extend dorsally from the brain neuropil of the developing larva (Fig. 2b, b’). At late stage 4, neurites with 5HT-LIR extend along the subesophageal commissure and posteriorly through the main connectives of the ventral nerve cord (Fig. 2e, e”; Additional file 2b, b”). One to two pairs of neuronal cell bodies with 5HT-LIR are present alongside the subesophageal commissure (Fig. 2e, e”). Additional neurites with 5HT-LIR begin to appear that underlie the prototroch and telotroch in the ventral part of the animal (Fig. 2b”, e” open arrows). Several peripheral neurites that have aTUB-LIR also form in the anterior-third of the trunk, just posterior to the prototroch (Additional file 1b, b’). Finally, a few ciliated cells positioned posterior to the mouth along the ventral midline are visible with aTUB-LIR at the end of stage 4 (Additional file 2e). These cells are the beginning of the neurotroch (nt), a ciliated band that runs along the ventral midline of the larva.

The number of cells and neurites contained within the brain increases from stage 5 to 6. Although the number of neurites in the brain increases, the pattern seen with aTUB-LIR remains very similar across these stages (compare Fig. 2b, b’ with d, d’). By stage 6, the number of neurons in the brain with 5HT-LIR has increased to six (Fig. 2d, d”), and neurites with 5HT-LIR now underlie most of the prototroch and telotroch (Additional file 1c” open arrowheads). The anterior cells with aTUB-LIR (sc^ac+^) increase to three on each side, and they are clearly contained within the dorsal-medial, anterior brain by stage 6 (Fig. 2c, d’; Additional file 1f open arrowheads).

In the ventral nerve cord from stage 5 to 6, additional longitudinal connectives are added to give a final number of five (compare Fig. 2e – g and Additional file 2a – d). The two lateral, or outer, connectives (main connectives) form first during stage 4 and are initially generated by neurons in the brain. The medial, or innermost, connective (ventromedian connective) forms next during stage 5 and initially forms from anterior to posterior. Finally, the two mediolateral, or intermediate, connectives (paramedian connectives) form last, during stage 6 and initially form from anterior to posterior. At the beginning of stage 5, multiple medial, longitudinal neurites are visible by aTUB-LIR and 5HT-LIR (Fig. 2f – f”). These medial neurites likely condense to form the ventromedian connective by the end of stage 5 (Additional file 2c – c” closed arrowhead). At the beginning of stage 6, the paramedian connectives begin to form and are visible with aTUB-LIR (Additional file 2d, d’ open arrowheads). Outside of the ventral nerve cord in the trunk, two additional pairs of ventral-lateral, longitudinal neurites with aTUB-LIR extend along the anterior-posterior axis of the trunk during stage 5 (Fig. 2f, f’; closed arrows point to the right pair). Of note is that many stage 5 and early stage 6 animals have errant neurites (open arrow in Fig. 2f” and Additional file 2d,’ d”). Presumably, these neurites get pruned since the pattern at the end of stage 6 is fairly stereotypical, and errant neurites are not usually visible by this stage (Fig. 2g – g”).

Overall, the ventral nerve cord develops from anterior to posterior. This can be seen by the gradual appearance of several commissures and segmentally-iterated peripheral nerves with aTUB-LIR from stage 5 to 6 (Fig. 2f, f’, g, g’). In the animal in Fig. 2f’ (stage 5), six forming commissures in the ventral nerve cord and six forming peripheral nerves (one in each segment) are visible by aTUB-LIR. In the animal in Fig. 2g’ (stage 6), at least nine forming commissures and nine forming pairs of peripheral nerves (two in each segment) are present. For reference, the lines in Fig. 2f, f’ and g, g’ mark the posterior boundary of segments three and four as determined by the morphological arrangement of nuclei. The ganglia of the ventral nerve cord begin to be visible by nuclear labeling at stage 5 and form from anterior to posterior. Early stage 5 animals have approximately three forming ganglia that are visible with a nuclear stain (data not shown). The stage 5 animal in Fig. 2f has at least 6 forming ganglia while the stage 6 animals in Fig. 2g and Additional file 1e have at least nine forming ganglia. Soma with 5HT-LIR appear in the ventral nerve cord with an anterior to posterior progression during stage 6 (Fig. 2g”), indicating the differentiation of some cells within the ganglia.

Within the trunk from stage 5 to 6, there is a narrowing and extension of the body and ventral nerve cord. This can be seen by comparing the distance between the two main, or outer, connectives of the ventral nerve cord (visible by aTUB-LIR) at stages 4 and 5 (compare Fig. 2e, e’ with f, f’). This process continues through stage 6 (Additional file 2a – d) and is largely complete by stage 7. The cilia of the neurotroch, visible by aTUB-LIR, undergo a similar process during this time (Additional file 2e – g).

There are several other notable features that can be seen by the end of stage 6. Additional neurites with aTUB-LIR are present on the lateral and dorsal surfaces of the trunk (Additional file 1c, c’). By this stage, there are two pairs of ventral-lateral, longitudinal neurite bundles and one pair of dorsal-lateral, longitudinal neurite bundles (Additional file 1c, c’ closed arrows and data not shown). Superficial cells with 5HT-LIR, some likely sensory neurons, also start to be visible in the head ectoderm, anterior trunk ectoderm, and pygidium, which is the region posterior to the telotroch (Fig. 2g”, Additional file 1f closed arrowheads and data not shown). Finally, and of some interest, is the appearance of a cell in the pygidium with 5HT-LIR (5HT-LIR pygidial cell, S-PC; Additional file 1c’, g, g’ closed arrowhead), which is morphologically similar to a posterior cell with 5HT-LIR in other annelids (see Discussion).

### FMRFamide-like-immunoreactivity (FMRF-LIR) in early-stage *C. teleta* larvae (stages 4 – 6)

**Fig. 3:**
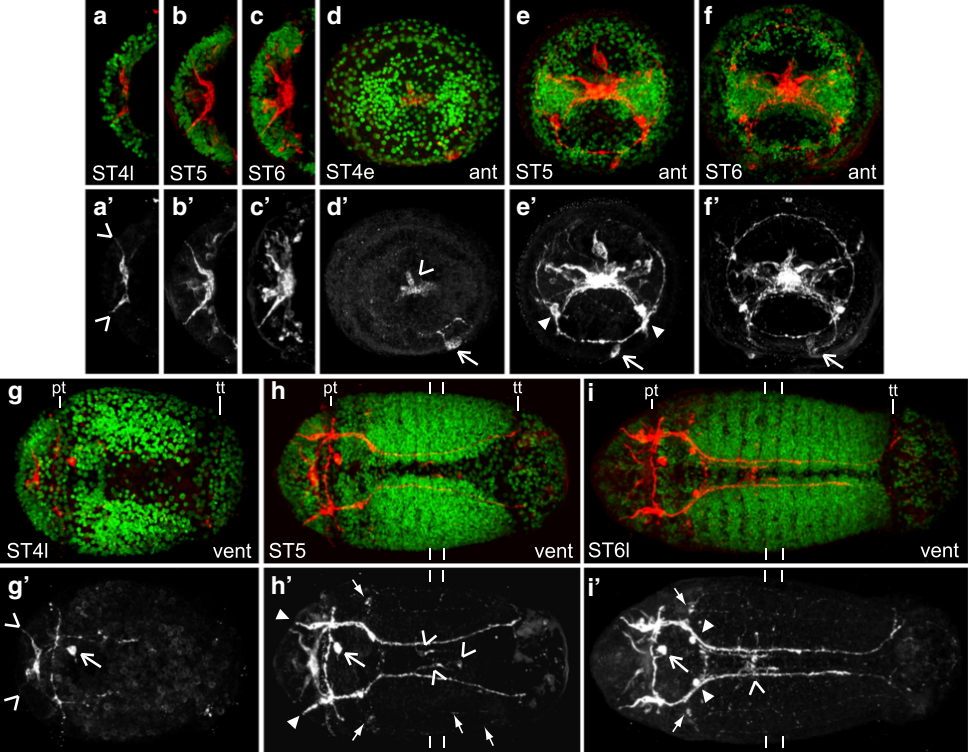
FMRF-LIR in early-stage *C. teleta* larvae (stages 4 – 6). Images are z-stack confocal images of larvae labeled with anti-FMRF (red) and Hoechst nuclear stain (green). Panels labeled with an apostrophe (*e.g.,* **a'**) are single-channel images of FMRF-LIR from the merged image without an apostrophe (*e.g.,* **a**). Panels **a** – **c** are cropped images of the brain. The scale bar in **g** is 50 μm, and all images are approximately the same scale. Open arrowheads in **a’** and **g’** indicate flask-shaped cells in the brain. The open arrowhead in **d’** points to a cluster of cells with 5HT-LIR in the brain. The open arrow in **d’**, **e’**, **f’** and **i’** points to F-AMC. Closed arrowheads in **e’** and **h’** mark flask-shaped cells in the head. The position of the prototroch (pt) and telotroch (tt) is indicated in **g**, **h** and **i**. Closed arrows in **h’** point to the right pair of ventral-lateral neurites that run from anterior to posterior. In **h’** and **i’**, open arrowheads mark neurons in the ventral nerve cord with FMRF-LIR, anterior closed arrows point to cells with FMRF-LIR positioned lateral to the mouth, and lines indicate the posterior boundary of segments four and five. In **i’**, two closed arrowheads point to cells near the mouth. Stage is indicated in the lower-left corner, and view is indicated in the lower-right corner, except for **a** – **c**, which are dorsal views. Anterior is to the left in all ventral and dorsal views, and ventral is down in all anterior views. ant, anterior; dors, dorsal; pt, prototroch; tt, telotroch; vent, ventral

Cross-reactive antibodies against the neurotransmitter FMRFamide allow visualization of a subset of neurons that are distinct from the ones with 5HT-LIR. Overall, the pattern of nervous system development visualized with FMRFamide-like-immunoreactivity (FMRF-LIR) from stages 4 – 6 is similar to that seen with aTUB-LIR and 5HT-LIR. Neurons and neurites with FMRF-LIR are first visible in the brain and around the mouth (Fig. 3a, d, g) and then later in the ventral nerve cord and periphery (Fig. 3h, i). Two differences from the pattern seen with 5HT-LIR are an asymmetric cell to the left of the mouth (Fig. 3d’ – i’ open arrow) and several flask-shaped cells with superficial extensions in the brain and head with FMRF-LIR (Fig. 3a – f). No other clearly asymmetric cells were identified in this study, and a flask-shaped morphology can be indicative of a sensory function in other animals ([47] and see Discussion).

Similar to the pattern seen for 5HT-LIR and aTUB-LIR, the first FMRF-LIR in *C. teleta* is visible early during stage 4. At this stage there are a few medial cells with FMRF-LIR in the developing brain (Fig. 3d, d’ open arrowhead) and a cell whose soma is positioned just anterior to the mouth on the left side of the larva (FMRF-LIR anterior mouth cell, F-AMC; Fig. 3d, d’ open arrow). This is the only asymmetric, unpaired neuron identified in this study. By late stage 4, two of the cells in the anterior neural ectoderm with FMRF-LIR adopt a flask-shaped morphology, each with a neurite that extends to the surface of the larva (Fig. 3a, a’, g, g’ open arrowheads). The other cells in the anterior ectoderm with FMRF-LIR are not flask-shaped. At this stage, F-AMC (Fig. 3g, g’ open arrow) sends a neurite in an anterior direction, which bifurcates at the prototroch and extends in both directions circumferentially along the anterior edge of prototroch. The F-AMC neurite only extends about half-way around the larva. There are also bilaterally-symmetric neurites with FMRF-LIR in the trunk that extend in a posterior direction and are in a similar position to the aTUB-LIR neurites (main connectives) in the ventral nerve cord at this stage (Fig. 3g, g’; compare with Fig. 2e, e’).

During stage 5, additional cell bodies and neurites with FMRF-LIR become visible in the brain, ventral nerve cord and periphery. In the developing brain, several more cells with FMRF-LIR become apparent (Fig. 3b, b’, e, e’). These cells include additional flask-shaped cells as well as cells that do not extend neurites to the surface. Outside of the brain, but in the head, an additional pair of flask-shaped cells with FMRF-LIR becomes visible on the ventral side of the larva, anterior to the prototroch (Fig. 3e, e’, h, h’ closed arrowheads). On the dorsal side of the larva, there are two cells with FMRF-LIR that are positioned along the midline. One of the cells is positioned anterior to the prototroch and extends neurites anteriorly into the brain and posteriorly along the midline towards the prototroch (Additional file 3a’ open arrowhead). The other cell is positioned posterior to the prototroch (FMRF-LIR dorsal midline cell, F-DMC; Additional file 3a’ closed arrowhead) and extends neurites anteriorly towards the prototroch and laterally around the circumference of the larva (similar to the early stage 6 animal in Additional file 3d, d’ closed arrowhead). F-DMC is also visible at late stage 4, but does not have neurites at this stage (data not shown).

Within the developing ventral nerve cord, three cells with FMRF-LIR that straddle the ventral midline in segments four to six become apparent at stage 5 (Fig. 3h, h’ open arrowheads). The posterior boundary of segments four and five as determined by nuclear labeling is indicated with lines in Fig. 3h, h’. These cells are positioned between the main connectives of the ventral nerve cord, which now extend posteriorly towards the telotroch and have FMRF-LIR (Fig. 3h, h’). Interestingly, the appearance of these cells in the middle of the developing ventral nerve cord deviates from the clearly anterior-to-posterior progression seen with other markers (aTUB-LIR, 5HT-LIR and *Ct-syt1*). Outside of the ventral nerve cord in the trunk, two cells with FMRF-LIR become apparent on the ventral side of the larva (Fig. 3h, h’ anterior closed arrows). Emanating from these cells are two pairs of ventral-lateral, longitudinal neurites with FMRF-LIR that extend along the anterior-posterior axis of the trunk (Fig. 3h, h’ posterior closed arrows point to the left pair). These neurites correspond in position to the two pairs of ventral-lateral, longitudinal neurites with aTUB-LIR that run parallel to the ventral nerve cord at stage 5 (Fig. 2f’ closed arrows).

During stage 6, new neurons and neurites with FMRF-LIR continue to be added in the brain and periphery and, to a lesser extent, the ventral nerve cord. The number of neurons in the brain with FMRF-LIR increases throughout stage 6 (Fig. 3c, c’, f, f’; Additional file 3b, b’, c, c’, e – g’). Also, within the head, there is a new pair of ventral-anterior cells (Additional file 3b’ closed arrow indicates the cell on the left side of the head) that are positioned ventral to the first pair of ventral-anterior flask-shaped cells that were visible at stage 5 (Fig. 3h’ closed arrowheads).

On the dorsal side of early stage 6 larvae, there are three cells with FMRF-LIR that are positioned just posterior to the prototroch (Additional file 3d, d’). The midline cell (F-DMC) extends a neurite anteriorly towards the prototroch (Additional file 3d’ closed arrowhead). The other two dorsal cells (Additional file 3d’ open arrowheads) extend neurites towards the prototroch and appear to be connected to F-DMC. By mid- to late-stage 6, there are five dorsal neurons (Additional file 3e’, f’ closed and open arrowheads) that send neurites into a ring that extends the full circumference of the prototroch (Fig. 3f’; Additional file 3b’, e’, f’ anterior-most open arrow). The dorsal trunk neurons also extend neurites into a circumferential ring around the dorsal-anterior trunk (Additional file 3f’ mid-anterior open arrow) and longitudinally towards the telotroch (Additional file 3c’, e’, f’ closed arrows). Neurites with FMRF-LIR also extend most of the way around the telotroch (Additional file 3b’, c’, f’ posterior-most open arrow).

On the ventral side of the larval trunk at stage 6, there are also a few new neurons and neurites with FMRF-LIR. Near the mouth, two cells positioned along the circumesophageal connective are visible with FMRF-LIR (Fig. 3i, i’ closed arrowheads). The pair of ventral-lateral neurons (Fig. 3i’ closed arrows) that send neurites into the circumesophageal connectives and longitudinally along the ventral-lateral side of the trunk are still present. F-AMC also remains visible through stage 6 (Fig. 3d’ – i’ open arrow). Within the ventral nerve cord, the connectives are now positioned closer to the midline (Fig. 3i, i’). There is a single commissure in the ventral nerve cord, likely the subesophageal commissure, with FMRF-LIR that is positioned posterior to the mouth (Fig. 3i, i’). Two of the ventral midline cells are now localized to segment four and appear to extend neurites along the forming paramedian connectives of the ventral nerve cord (Fig. 3i’ open arrowhead). Additional soma with FMRF-LIR were not detected in the ventral nerve cord (Fig. 3i; Additional file 3b – c’), even though several ganglia are clearly visible with nuclear labeling and contain cells with 5HT-LIR at this stage (Fig. 2g, g”). There are two additional neurons with FMRF-LIR at the posterior end of the main connectives, at the same anterior-posterior position as the telotroch (Additional file 3b’ closed arrowhead points to the left one). Finally, within the pygidium at stages 5 and 6, there is FMRF-LIR (*e.g.*, Additional file 3c’), but it is not clear if this staining corresponds to cells.

### *Ct-syt1* homolog expression in early-stage larvae (stages 4 – 6)

**Fig. 4:**
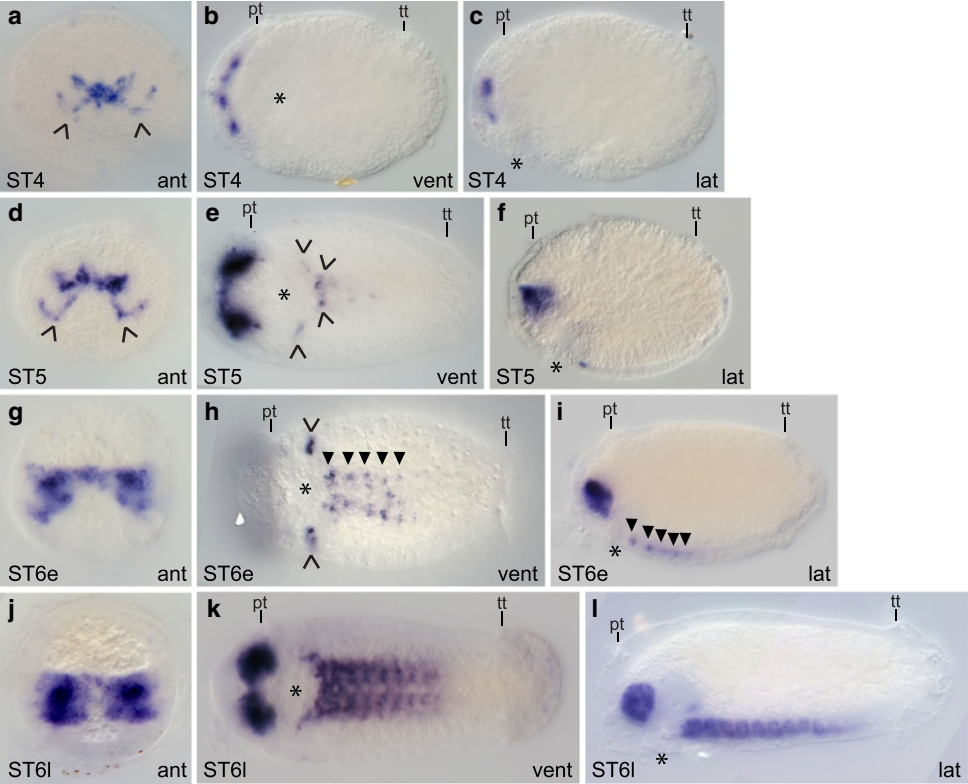
*Ct-syt1* expression in early-stage stage *C. teleta* larvae (stages 4 – 6). Panels show DIC images of animals after whole mount in situ hybridization for the *synaptotagmin1* homolog *Ct-syt1*. In panels **d**, **g**, **j** and **k**, DIC images from multiple focal planes were merged in Helicon Focus to show more of the *Ct-syt1* expression pattern. Open arrowheads in A and D indicate *Ct-syt1* ^+^ cells along the ventral edge of the developing brain. The position of the prototroch (pt) and telotroch (tt) is indicated in **b**, **c**, **e**, **f**, **h**, **i**, **k** and **l**. Open arrowheads in E mark *Ct-syt1* ^+^ cells around the mouth. Closed arrowheads in H and I point to *Ct-syt1* ^+^ cells in the ganglia of the ventral nerve cord. Asterisks mark the position of the mouth. Stage is indicated in the lower-left corner, and view is indicated in the lower-right corner. All lateral views are of the left side. Anterior is to the left in all ventral, dorsal and lateral views, and ventral is down in all anterior views. ant, anterior; dors, dorsal; lat, lateral; pt, prototroch; tt, telotroch; vent, ventral

*Synaptotagmin 1* (*syt1*) homologs are expressed in most if not all terminally differentiated neurons in many animals [40–42], which allows visualization of mature neurons within the developing nervous system. *Capitella teleta* has one *synaptotagmin 1* homolog, *Ct-syt1*. In general, *Ct-syt1* expression progresses from anterior to posterior and begins in the central nervous system. Expression begins at stage 4 in a few cells in the developing brain (Fig. 4a – c), and during stage 5 and early stage 6 expands to a few cells around the mouth and in the ventral nerve cord (Fig. 4d – i). By the end of stage 6, most cells in the brain and anterior three-quarters of the ventral nerve cord ganglia express *Ct-syt1* (Fig. 4j – l).

*Ct-syt1* is first expressed consistently at stage 4 in several basally-localized cells within the forming brain (Fig. 4a – c). Prior to stage 4, we were able to detect *Ct-syt1* expression in the forming brain in only two out of 45 stage 3 embryos, and these animals may have already been transitioning to stage 4. By stage 5, more cells in the developing brain express *Ct-syt1* (Fig. 4d, f), and, similar to stage 4, *Ct-syt1* expression is in a subset of brain cells that appear to lie along the ventral edge of the forming brain (Fig. 4a, d open arrowheads). *Ct-syt1* expression is also visible in the trunk at stage 5, in at least six cells positioned lateral and posterior to the mouth (Fig. 4e open arrowheads), near the circumesophageal connectives and subesophageal commissure.

Early during stage 6, the two lobes of the brain become more well-defined and many cells within the developing brain express *Ct-syt1* (Fig. 4g, i). *Ct-syt1* expression in the brain continues to increase as stage 6 proceeds (Fig. 4j – l). Within the trunk at early stage 6, *Ct-syt1* is expressed in a few cells on either side of the mouth (Fig. 4h open arrowheads) and within the anterior three to five forming ganglia of the ventral nerve cord (Fig. 4h, i closed arrowheads). By late stage 6, *Ct-syt1* is expressed in most but not all of the forming ganglia in the ventral nerve cord. For example, the animal in Fig. 4k expresses *Ct-syt1* in the nine anterior-most ganglia out of 10 total while the animal in Fig. 4l expresses *Ct-syt1* in the 10 anterior-most ganglia out of 11 total. The number of ganglia present was determined by nuclear stain (data not shown).

### aTUB-LIR and 5HT-LIR in late-stage *C. teleta* larvae (stages 7 – 9)

**Fig. 5:**
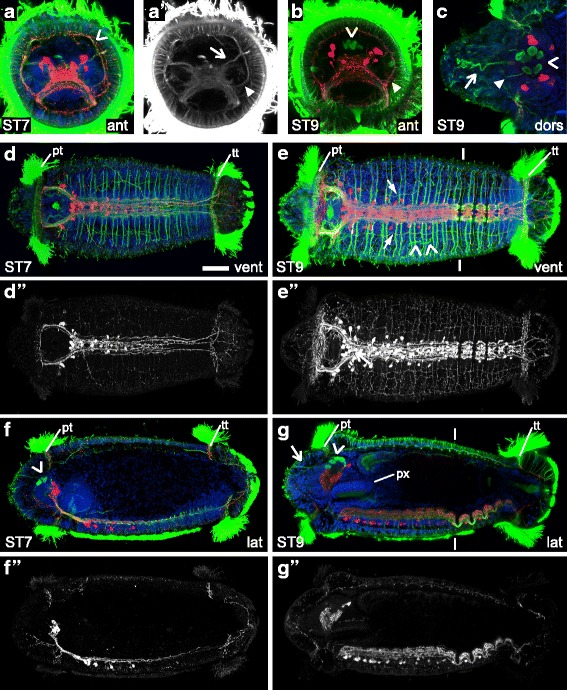
aTUB-LIR and 5HT-LIR in late-stage *C. teleta* larvae (stages 7 – 9). Images are z-stack confocal images of larvae labeled with anti-acetylated-α-tubulin (green), anti-serotonin (red) and TO-PRO-3 nuclear stain (blue). Panels labeled with an apostrophe (*e.g.,* **a'**) are single-channel images of either aTUB-LIR (’) or 5HT-LIR (”) from the merged image without an apostrophe (*e.g.,* **a**) except where otherwise noted. The scale bar in **a** is 50 μm, and all images are to approximately the same scale except where otherwise noted. **c** is a cropped, 2.1x magnified image of the brain. In **a**, an open arrowhead points to the prototrochal nerve ring. The open arrow in **a’** points to a dorsal bundle of neurites that extend from the brain neuropil. In **a’** and **b**, a closed arrowhead marks the position of a dorsal split in the circumesophageal connective. An open arrowhead in **b**, **c**, **f** and **g** points to the sc^ac+^. The open arrow in **c** and **g** indicates a bundle of neurites that extend to the surface. The position of the prototroch (pt) and telotroch (tt) is indicated in **d**, **e**, **f**, and **g**. In **e**, the open arrowheads mark the right pair of metanephridia, and the closed arrows point to ventral-lateral, longitudinal neurites. Lines in **e** and **g** indicate the approximate thoracic-abdominal boundary. In **g**, the dorsal pad of the pharynx is labeled with a “px”. Stage is indicated in the lower-left corner, and view is indicated in the lower-right corner. All lateral views are of the left side. Anterior is to the left in all ventral, dorsal and lateral views, and ventral is down in all anterior views. ant, anterior; dors, dorsal; lat, lateral; pt, prototroch; px, pharynx; tt, telotroch; vent, ventral

Stage 7 is marked by formation of the chaetae and, by the end of stage 9, *C. teleta* larvae are competent to metamorphose. We examined nervous system development at stages 7, 8 and 9, but only report the patterns for stages 7 and 9 because stage 8 was very similar to both. Overall, the brain does not change very much (Fig. 5a – b) while the ventral nerve cord continues to add ganglia and neurites (Fig. 5d – g). The number of cell bodies and neurites with 5HT-LIR in the ventral nerve cord dramatically increases from stage 7 to 9 (Fig. 5d”, e”). By stage 9, the thoracic and abdominal portions of the ventral nerve cord and the associated peripheral nerves are discernibly different, there are many more neurites with aTUB-LIR and 5HT-LIR in the periphery of the trunk (Fig. 5e, e”), and neurites with aTUB-LIR in the stomatogastric nervous system are visible.

From stage 7 to stage 9, the brain increases in size slightly and the number of neurons with 5HT-LIR in the brain goes from 8 to 14. This number is somewhat variable, and individual stage 8 and 9 animals have between 10 and 14 neurons with 5HT-LIR. Cell bodies with 5HT-LIR are bilaterally symmetric and are dorsally and posteriorly positioned in the brain (Fig. 5a – c). There are a large number of neurites with 5HT-LIR in the brain neuropil (Fig. 5a, b, f, f”, g, g”). Within the brain neuropil, clear dorsal and ventral commissures, or tracts, as is seen in many adult polychaetes [48], are not visible by aTUB-LIR or 5HT-LIR (Fig. 5a, a’, b).

Other features visible with aTUB-LIR in the head at stages 7 and 9 include bundles of neurites that extend dorsally from the circumesophageal connectives (Fig. 5a’, b closed arrowhead points to the left bundle) and project to the ring underlying the prototroch. There is also a pair of neurite bundles that extend dorsally from the brain neuropil (Fig. 5a’ open arrow points to the left bundle). Neurites with aTUB-LIR and 5HT-LIR are also visible in a ring underlying the prototroch (Fig. 5a open arrowhead; Fig. 5a’, b). The sc^ac+^ are still clearly visible in the dorsal-medial brain at stages 7 and 9 (Fig. 5b, c, f, g open arrowhead), and they extend projections to the surface of the epidermis. One of these projections is indicated with a closed arrowhead in Fig. 5c. There is also a bundle of neurites with aTUB-LIR that extend from the brain to the epidermis in the dorsal-medial head (Fig. 5c, g open arrow). Another notable feature in the head are the nuchal organs, ciliated chemosensory organs found in most polychaetes [49], which can be seen in *C. teleta* by aTUB-LIR just anterior to the prototroch at stages 8 and 9 (Additional file 4c open arrowheads).

Several changes can be seen in the larval trunk from stages 7 – 9. The ganglia in the ventral nerve cord become larger and more discrete (Additional file 4f, g) and increase in number from 10 to 13 (Fig. 5d – g). By this stage, there is also a 14th ganglion forming. There are more soma in the ventral nerve cord with 5HT-LIR compared with earlier stages. These soma appear from anterior to posterior, are present in all 13 ganglia by stage 9 (compare Fig. 5d, d”, f, f” with Fig. 5e, e”, g, g”), and are not obviously bilaterally symmetric (Fig. 5e”). The number of neurites in the ventral nerve cord (aTUB-LIR and 5HT-LIR) also increases, making the anterior and posterior commissures in each segment and the five main longitudinal connectives more difficult to discern as discrete nerves (Fig. 5e, e”; Additional file 4f, f’, g, g’). The approximate thoracic-abdominal boundary is marked with lines in Fig. 5e, g. At stage 9, the thoracic (1 – 9) and abdominal (10 – 13) ganglia are different from each other (Fig. 5e, e”, g, g”; compare Additional file 4f, f’ and g, g’) in their dorsal-ventral position, size of ganglia, number of neurons with 5HT-LIR and the pattern of commissures and connectives (discussed more in the juvenile results section).

Paired main peripheral nerves (two per segment) extend from the ventral nerve cord in the anterior 10 or 11 segments at stage 7 (Fig. 5d, Additional file 4a) and in all 13 fully-formed larval segments plus the 14th forming segment at stage 9 (Fig. 5e, Additional file 4b). One pair of main peripheral nerves in segment 11 is bracketed in Additional file 4b. The paired main peripheral nerves appear to run along the basal side of the epidermis. The anterior of the two main nerves is positioned in the middle of each ganglion, and the posterior is positioned at the posterior edge of each ganglion, near the circular muscle fibers (data not shown). The posterior main nerve abuts the posterior boundary of each segment, which was determined by tissue morphology and the position of nuclei. Within the thorax, the paired main peripheral nerves at the posterior edge of each segment completely encircle the larva, while the anterior of the two paired main peripheral nerves does not extend all the way around the dorsal side of the larva (Additional file 4e, brackets mark one pair of main peripheral nerves). Within the abdomen, most of the paired main peripheral nerves do not completely extend around the dorsal side of the larvae. On the ventral and lateral sides of each abdominal segment, there is a third, thin minor peripheral nerve underlying the epidermis and positioned in the middle of each abdominal segment. This minor nerve is positioned between the posterior main peripheral nerve in the segment to the anterior and the anterior main peripheral nerve in the same segment (Fig. 5e, Additional file 4b). A minor peripheral nerve in segment 11 is indicated with an asterisk in Additional file 4b.

The two pairs of ventral-lateral longitudinal neurite bundles (the ventral pair that runs along the outside of the ventral nerve cord is indicated in Fig. 5e with closed arrows) and the pair of dorsal-lateral longitudinal neurite bundles that runs along the dorsal side of the larva (Additional file 4e closed arrows) are still present. All three pairs of neurite bundles are visible with aTUB-LIR and the most ventral pair is visible with 5HT-LIR by stage 9. In addition to these longitudinal neurites, unpaired peripheral neurites with aTUB-LIR and 5HT-LIR can be seen throughout the epidermis in the trunk, pygidium and underlying the telotroch (Fig. 5d, d”, e, e”; Additional file 4a, a”, b, b”). Neurites innervating the stomatogastric system can also be seen by stage 9. Two pairs of neurite bundles with aTUB-LIR (one pair anterior to the mouth and one pair posterior to the mouth) extend from the circumesophageal connectives (Fig. 5e; Additional file 4d open arrowheads point to the two left bundles) and project along the lateral sides of the dorsal pad of the pharynx (Additional file 4c open arrow points to the right bundle). In *C. teleta*, the pharynx is part of the foregut and the dorsal side (dorsal pad) is relatively thick when compared with the ventral side [50, 51]. For reference, the dorsal pad of the pharynx is labeled with a “px” in Fig. 5g and Additional file 4c. Neurites with 5HT-LIR are found throughout the dorsal pad of the pharynx (data not shown). Also visible by aTUB-LIR at stage 9 are four metanephridia [52–54] that span segments 5 – 7 (Fig. 5e open arrowheads point to the right pair).

### FMRF-LIR in late-stage *C. teleta* larvae (stages 7 – 9)

**Fig. 6:**
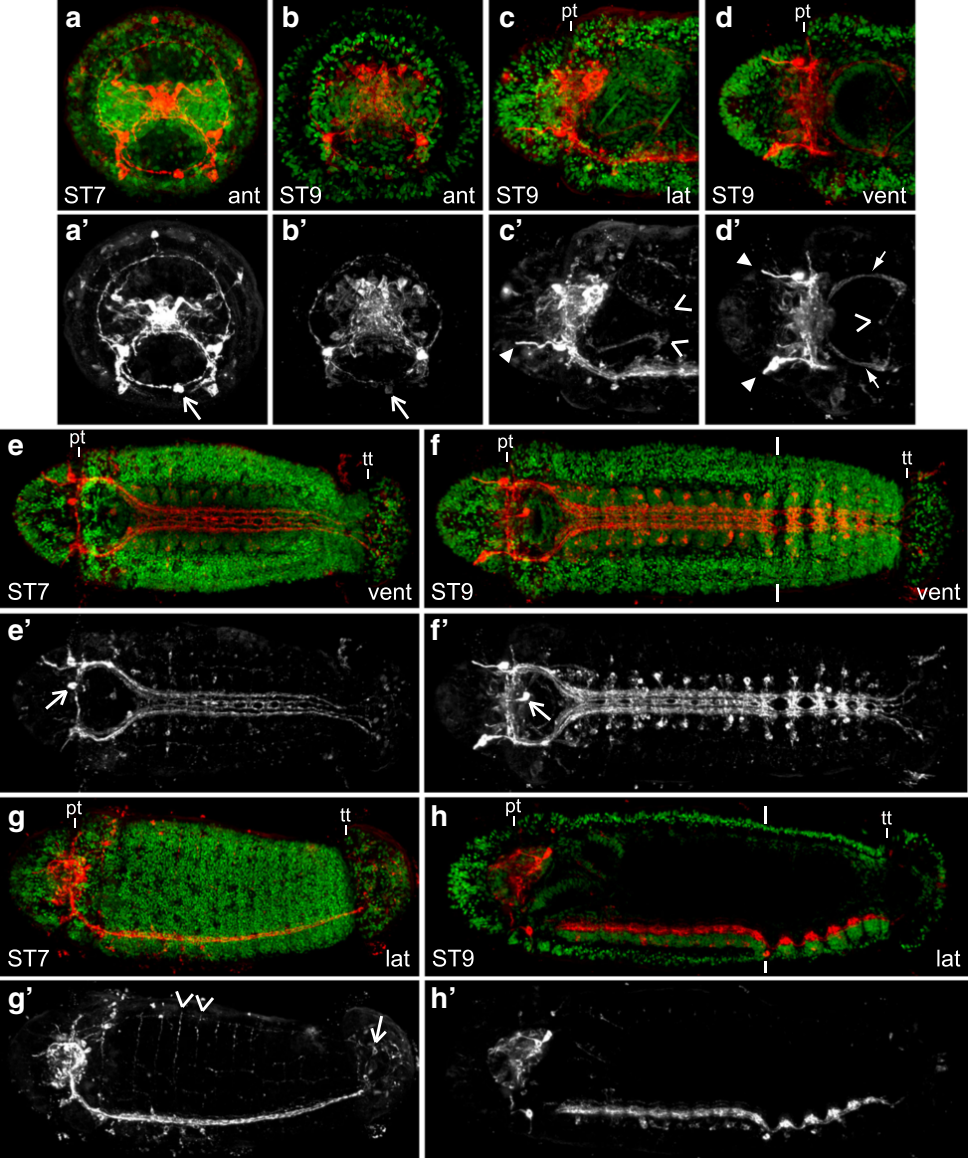
FMRF-LIR in late-stage *C. teleta* larvae (stages 7 – 9). Images are z-stack confocal images of larvae labeled with anti-FMRF (red) and Hoechst nuclear stain (green). Panels labeled with an apostrophe (*e.g.,* **a'**) are single-channel images of FMRF-LIR from the merged image without an apostrophe (*e.g.,* **a**). Panels **c** and **d** are cropped images of the head. The open arrow in **a’**, **b’**, **e’**, **f’** points to F-AMC. The position of the prototroch (pt) is indicated in **c**, **d**, **e**, **f**, **g** and **h**, while the telotroch (tt) is indicated in **e**, **f**, **g** and **h**. Open arrowheads in **c’** mark left-lateral clusters of neurons near the pharynx. Closed arrowheads in **c’** and **d’** point to flask-shaped cells that are positioned on the ventral side of the larva, just anterior to the prototroch. In **d’**, the open arrowhead points to a single cell with FMRF-LIR that is just ventral to the pharynx, and the closed arrows point to neurite bundles extending from the dorsal cluster of neurons with FMRF-LIR near the pharynx. In G’ open arrowheads indicate two of the segmentally-iterated dorsal trunk neurites positioned between the mesoderm and endoderm, and the open arrow points to a posterior cell in the pygidium. Stage is indicated in the lower-left corner, and view is indicated in the lower-right corner. All lateral views are of the left side. Anterior is to the left in all ventral and lateral views, and ventral is down in all anterior views. ant, anterior; lat, lateral; pt, prototroch; tt, telotroch; vent, ventral

As *C. teleta* larvae progress towards being competent to metamorphose (stages 7 – 9), more neurons with FMRF-LIR appear in the brain (Fig. 6a – d, g, h) and ganglia of the ventral nerve cord (Fig. 6e, f, h). Several commissures and the paramedian connectives in the ventral nerve cord are visible with FMRF-LIR (Fig. 6e, f). By stage 9, extensive FMRF-LIR is visible in cell bodies and neurites in the stomatogastric nervous system (Fig. 6c, c, d, d), the asymmetrically-localized cell by the mouth (F-AMC, Fig. 6b’, f’ open arrow) and the flask-shaped cells in the head.

At stage 7, the brain has many cells with FMRF-LIR, including several flask-shaped cells and many non-flask-shaped cells (Fig. 6a, a’, g, g’, Additional file 5a, a’). Within the trunk, cells with FMRF-LIR begin to appear in the ganglia of the ventral nerve cord in an anterior to posterior progression (Fig. 6e, e’). For example, the larva in Fig. 6e has FMRF-LIR in a small subset of cells in the seven anterior-most ganglia. Likewise, many more neurites with FMRF-LIR are visible in the two pairs of main and paramedian connectives of the ventral nerve cord, and there are at least nine ganglia whose commissures have FMRF-LIR (Fig. 6e, e’). The appearance of FMRF-LIR in the commissures and connectives of the ventral nerve cord is delayed relative to their formation since several commissures and all five connectives (two main, two paramedian and one median) are visible with aTUB-LIR by stage 6 (Fig. 2g, g’). There are also several new cells with FMRF-LIR in the pygidium, posterior to the telotroch (Fig. 6g' open arrow points to one soma).

On the dorsal side of stage 7 larvae, new cells with FMRF-LIR appear in a somewhat disorganized pattern in the anterior region of the trunk, just posterior to the prototroch (data not shown). In addition, segmentally-iterated, circumferential neurites with FMRF-LIR appear on the dorsal side of the larval trunk and are positioned basal to the epidermis (Fig. 6g', g’; open arrowheads point to two in G’). These neurites largely co-localize (data not shown) in position with the main peripheral nerves seen with aTUB-LIR (*e.g.,* Additional file 4a). However, the circumferential neurites with FMRF-LIR form from dorsal to ventral versus the main peripheral nerves, which form from ventral to dorsal.

By stage 9, there are many neurons in the brain with FMRF-LIR (Fig. 6b, b’, c, c’, h, h’; Additional file 5b – e). Neurites with FMRF-LIR in the brain neuropil are split into a ventral-anterior tract (Additional file 5d’ closed arrowhead) and a dorsal-posterior tract (Additional file 5d’ open arrowhead). This is in contrast to the overall pattern seen within the brain neuropil with aTUB-LIR, which is not split into different tracts (*e.g.,* Fig. 5a’). There are also cells with FMRF-LIR in the anterior head epidermis (data not shown), and the ventral-anterior flask-shaped cells that were first visible at stage 5 (Fig. 3e’, h’ closed arrowheads) are still present (Fig. 6c’, d’, closed arrowheads). F-AMC remains visible throughout larval development (Fig. 6b, b’, f, f’ open arrow), and a bilaterally symmetric partner on the right-anterior side of the mouth region was never detected.

Within the trunk at stage 9, most of the ganglia in the ventral nerve cord have FMRF-LIR in subsets of cells and in the commissures. For example, the larvae in Fig. 6f and h have cell bodies and commissures with FMRF-LIR in all but the most posterior, not-fully-formed ganglion (13 out of 14 ganglia). The soma of cells in the ventral nerve cord with FMRF-LIR are positioned laterally, dorsally and ventrally within each ganglion (Fig. 6f, f’, h, h’, Additional file 5b’). Within the dorsal trunk, there is a pair of longitudinal neurites with FMRF-LIR (Additional file 5e’ closed arrows), and the segmentally-iterated circumferential fibers are still visible (Additional file 5b’, e’ open arrowheads point to two).

The stomatogastric nervous system begins to display FMRF-LIR starting at stage 8 and continues into stage 9 (Fig. 6c’, d’). There are four clusters of cells with FMRF-LIR that surround the pharynx. In Fig. 6c and c’ two clusters of neurons with FMRF-LIR on the left side of the larva can be seen, one on the dorsal side and one on the ventral side of the pharynx (indicated with open arrowheads). The dorsal pair of clustered cells extends neurites anteriorly along the dorsal-lateral edge of the dorsal pad of the pharynx (Fig. 6d, d’ closed arrows). These neurites join bundles of neurites extending anteriorly and dorsally from the ventral nerve cord (not shown). There is also a thin pair of neurites with FMRF-LIR that extend towards a medial cell with FMRF-LIR, which is just ventral to the pharynx (Fig. 6d’ open arrowhead).

### *Ct-syt1* homolog expression in late-stage larvae (stages 7 – 9)

**Fig. 7:**
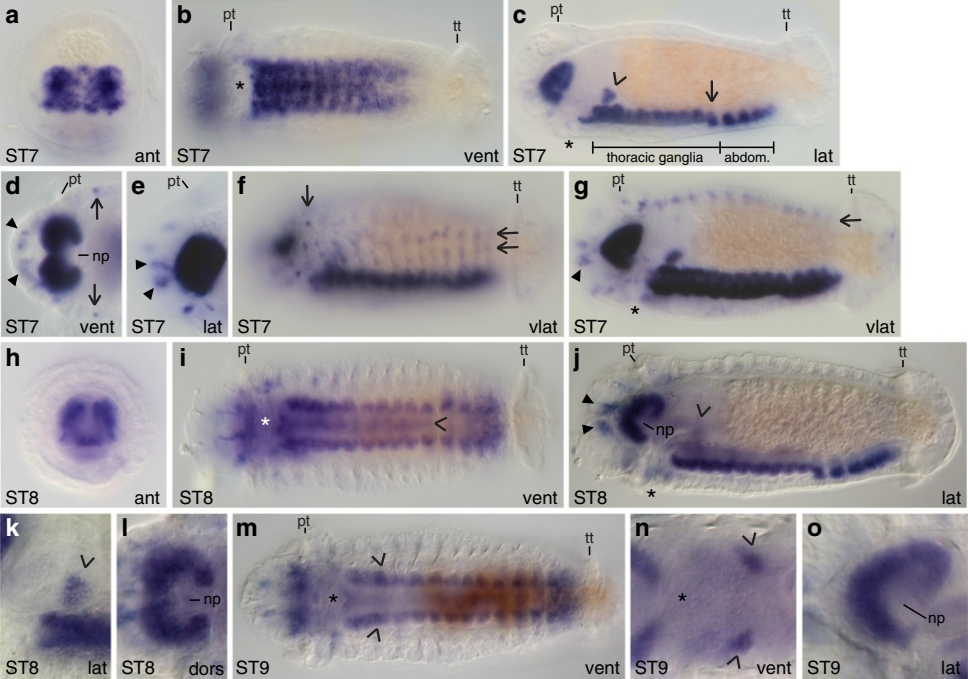
*Ct-syt1* expression in late-stage stage *C. teleta* larvae (stages 7 – 9). Panels show DIC images of animals after whole mount in situ hybridization for *Ct-syt1*. Panels **d** and **e** are cropped images of the head. All panels are to the same scale unless otherwise noted. Panels **l** and **o** are cropped, 2x magnified images of the brain. Panels **k** and **n** are cropped, 2x magnified images of the pharynx and correspond to panels **j** and **m**, respectively, but are different focal planes. The position of the prototroch (pt) and/or telotroch (tt) are indicated in **b** – **g**, **i**, **j** and **m**. The open arrowheads in **j** and **m** point to the position of the *Ct-syt* ^+^ cells in **k** and **n**, respectively. Open arrowheads in **c**, **j**, **k** and **n** indicate *Ct-syt1* ^+^ cells near the pharynx. In **c**, the boundaries of the thoracic and abdominal (abdom.) ganglia are indicated, and an open arrow points to the 9th thoracic ganglion. The brain neuropil (np) is indicated in **d**, **j**, **l** and **o**. Closed arrowheads in **d**, **e**, **g** and **j** point to *Ct-syt1* ^+^ cells in the head. Open arrows in **d** and **f** point to a circumferential row of *Ct-syt1* ^+^ cells in the head epidermis, just posterior to the brain. Open arrows in **f** and **g** point to longitudinal rows of *Ct-syt1* ^+^ cells in the trunk epidermis. The open arrowhead in **i** points to clusters of *Ct-syt1* ^+^ midline cells within the ventral nerve cord. An asterisk marks the approximate position of the mouth. Stage is indicated in the lower-left corner, and view is indicated in the lower-right corner. **c** and **f** are reflected right-lateral views. Anterior is to the left in all ventral, dorsal and lateral views, and ventral is down in all anterior views. ant, anterior; dors, dorsal; lat, lateral; pt, prototroch; tt, telotroch; vent, ventral; vlat, ventral-lateral

In general, the pattern of *Ct-syt1* expression from stage 7 to 9 does not appear to change very much in the brain (Fig. 7a, c, d, g, h, j, l, o). The expression pattern in the ventral nerve cord extends more posteriorly such that all 13 ganglia in the ventral nerve cord express *Ct-syt1* (compare Fig. 7b, i, m and c, j). Many peripheral neurons throughout the body (Fig. 7d, e, f, g, j closed arrowheads and open arrows) and neurons in the stomatogastric ganglia (Fig. 7c, k, n open arrowhead) also begin to express *Ct-syt1* at late larval stages.

At stage 7, *Ct-syt1* is expressed throughout the bilobed brain (Fig. 7a, d) but is absent from the prominent neuropil (Fig. 7d np). Approximately 10 – 12 ganglia in the ventral nerve cord express *Ct-syt1* (Fig. 7b, c). The abdominal ganglia are positioned more superficially than the eight anterior-most thoracic ganglia (Fig. 7c), while the ninth thoracic ganglion occupies an intermediate position between the two groups (Fig. 7c open arrow). There are also two clusters of *Ct-syt1*^+^ cells on the left and right sides of the forming pharynx (one is indicated in Fig. 7c with an open arrowhead), which are likely stomatogastric ganglia. In addition, several *Ct-syt1*^+^ cells, presumably sensory neurons, are present in the head epidermis (Fig. 7d, e closed arrowheads point to a few cells) and in a row encircling the body just posterior to the prototroch (Fig. 7d, f vertical open arrows). Two ventral-lateral, longitudinal rows (Fig. 7f horizontal open arrows) and one dorsal-lateral, longitudinal row (Fig. 7g horizontal open arrow) of *Ct-syt1*^+^ cells, likely peripheral neurons, are present on each side of the trunk.

At stages 8 and 9, *Ct-syt1* expression in the brain is very similar to stage 7 (Fig. 7h, j, l, o), although the brain looks more compact when seen from an anterior view (Fig. 7h). In addition, all 13 ganglia in the ventral nerve cord express *Ct-syt1* (Fig. 7i, j, m). *Ct-syt1* expression, which is found within the soma of neurons, is localized to the ventral half and outer edges of the nerve cord (Fig. 7i, j, m). There are also a small number of *Ct-syt1*^+^ soma along the dorsal midline of the nerve cord (Fig. 7i open arrowhead). The two neuropils in the ventral nerve cord run along the dorsal-medial sides of the cord. By stage 8, the esophagus and pharynx can be clearly distinguished [50]. The putative stomatogastric ganglia are visible at stages 8 and 9 as clusters of *Ct-syt1*^+^ cells lateral to the posterior edge of the pharynx (Fig. 7k, n open arrowheads) but ventral to the esophagus. The approximate position of the stomatogastric ganglia are indicated in Fig. 7j and m with open arrowheads, although they are not visible at the focal plane shown. *Ct-syt1* expression is also maintained in single and small clusters of cells in the epidermis at late larval stages (Fig. 7j closed arrowheads and data not shown).

### aTUB-LIR, 5HT-LIR, FMRF-LIR and *Ct-syt1* expression in *C. teleta* juveniles

**Fig. 8:**
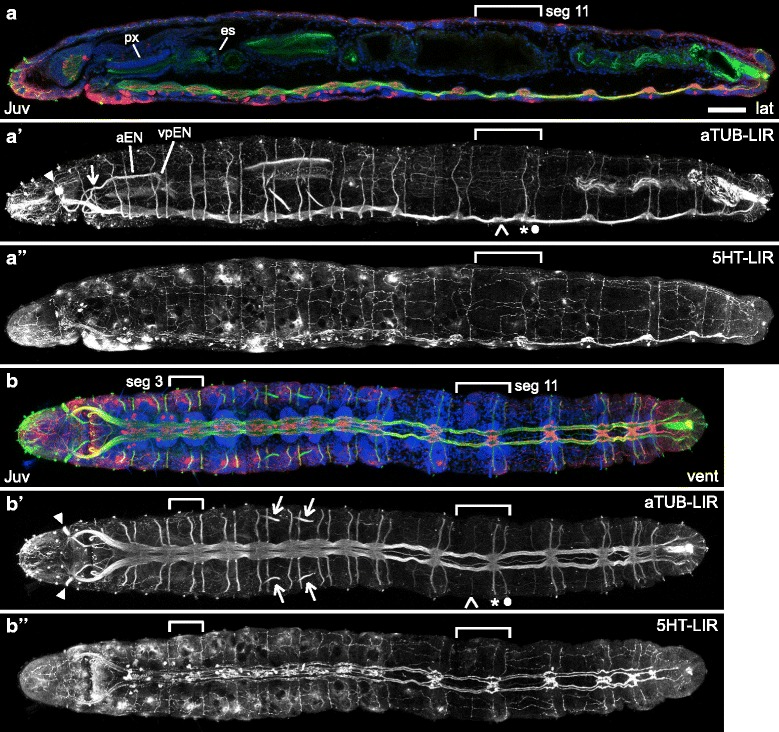
aTUB-LIR and 5HT-LIR in 7-day old *C. teleta* juveniles. Images are z-stack confocal images of 7-day old juveniles labeled with anti-acetylated-α-tubulin (green), anti-serotonin (red) and TO-PRO-3 nuclear stain (blue). Panels with the same letter are different focal planes of the same animal. Merged images (**a**, **b**) are z-stacks that begin below the surface, while the single-channel images (**a’**, **a”**, **b’**, **b”**) begin at the surface. **a’** and **b’** are single-channel images of anti-acetylated-α-tubulin labeling while **a”** and **b”** are single-channel images of anti-serotonin labeling. The scale bar in A is 50 μm, and all images are to approximately the same scale. The pharynx (px) and esophagus (es) are labeled in **a**. Brackets in **a** – **a”** and **b** – **b”** mark the boundaries of segment (seg) 11. Segment 3 is also bracketed in **b** – **b”**. In **a’** and **b’**, an open arrowhead marks the minor abdominal peripheral nerve in segment 11, and the asterisk and filled circle indicate the two main peripheral nerves in segment 11. In **a’**, a closed arrowhead points to the left nuchal organ, an open arrow indicates the first branchpoint in the left anterior enteric nerve (aEN), and the left ventral-posterior enteric nerve (vpEN) is indicated. In **b’**, closed arrowheads point to the nuchal organs, and open arrows indicate the metanephridia. Stage is indicated in the lower-left corner, and view is indicated in the lower-right corner. All lateral views are of the left side. Anterior is to the left in all views. es, esophagus; lat, lateral; px, pharynx; vent, ventral

Competent stage 9 *C. teleta* larvae metamorphose into crawling juvenile worms within a few hours of being exposed to an appropriate cue and begin feeding within a day. Metamorphosis in *C. teleta* is not a dramatic process, and some of the most noticeable changes are loss of the ciliary bands and body elongation (Fig. 8a, b). Most features of the nervous system in stage 9 larvae are present in juveniles (*e.g.*, compare Fig. 8a with 5G and Fig. 8b with 5E), suggesting that the nervous system that is formed during larval development is not significantly lost or rearranged at metamorphosis and that brain and ventral nerve cord are shared between the larva and adult.

**Fig. 9:**
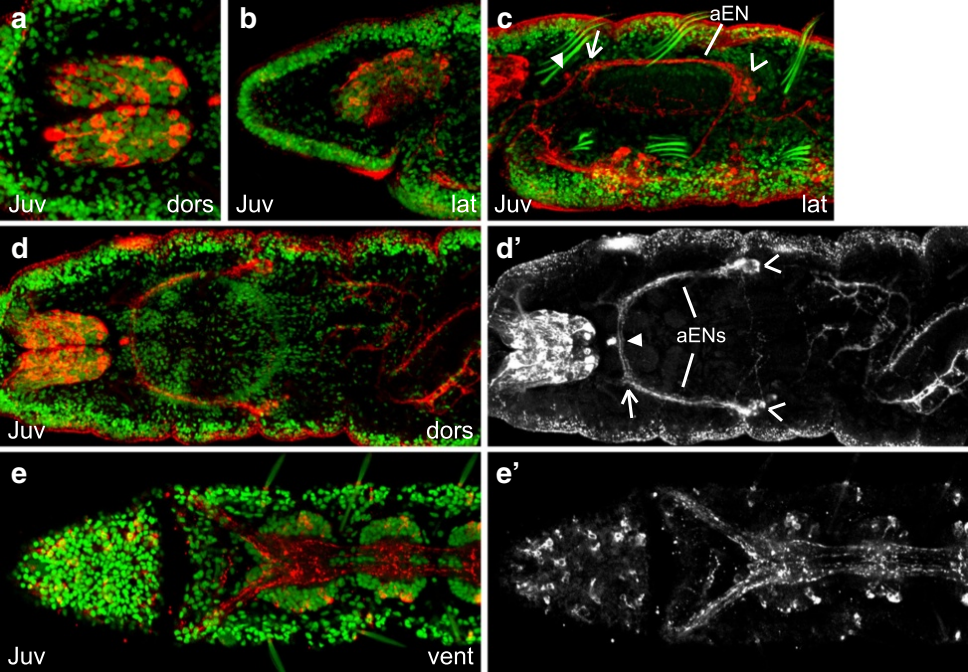
FMRF-LIR in 7-day old *C. teleta* juveniles. Images are z-stack confocal images of 7-day old juveniles labeled with anti-FMRF (red) and Hoechst nuclear stain (green). Panels labeled with an apostrophe (*e.g.,* **a'**) are single-channel images of FMRF-LIR from the merged image without an apostrophe (*e.g.,* **a**). All panels are to the same scale unless otherwise noted. **a** is a cropped, 1.25x magnified image of the brain. **b** is a cropped view of the brain; **c** is a cropped view of the pharynx; **d** is a cropped view of the brain, pharynx and anterior portion of the midgut; **e** is a cropped view of the head and first two ganglia in the ventral nerve cord. In **c** and **d’**, open arrowheads point to clusters of neurons with FMRF-LIR near the pharynx (stomatogastric ganglia), a closed arrowhead points to a single cell with FMRF-LIR that is anterior to the medial portion of the pharynx, the open arrow indicates the first branchpoint in the anterior enteric nerve (aEN). Stage is indicated in the lower-left corner, and view is indicated in the lower-right corner. All lateral views are of the left side. Anterior is to the left in all ventral, dorsal and lateral views, and ventral is down in all anterior views. ant, anterior; dors, dorsal; lat, lateral; vent, ventral

**Fig. 10:**
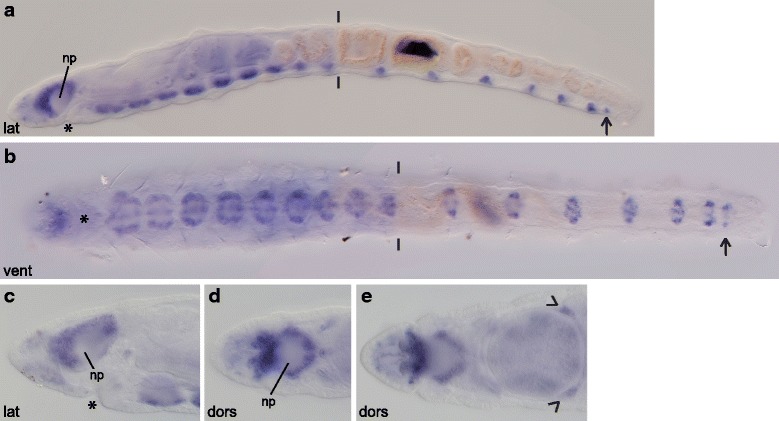
*Ct-syt1* expression in 7-day old *C. teleta* juveniles. Panels show DIC images of 7-day old juveniles after whole mount in situ hybridization for *Ct-syt1*. In panels **a** and **c**, DIC images from multiple focal planes were merged in Helicon Focus to show more of the *Ct-syt1* expression pattern. All panels are to the same scale unless otherwise noted. **c** is a cropped, 1.5x magnified image of the brain, **d** is a cropped, 1.7x magnified image of the brain, and **e** is a cropped, 1.7x magnified image of the brain and the pharynx. In **a** and **b**, the thoracic-abdominal boundary is indicated with two lines, and the open arrow points to a newly forming ganglion. The brain neuropil (np) is indicated in **a**, **c** and **d**. The open arrowheads in **e** indicate *Ct-syt1* ^+^ cells near the pharynx. An asterisk marks the approximate position of the mouth. View is indicated in the lower-left corner. All lateral views are of the left side. Anterior is to the left in all views. dors, dorsal; lat, lateral; vent, ventral

The brain in juveniles is roughly the same size and shape as in stage 9 larvae (compare Fig. 8a with Fig. 5g), and both juvenile and late-stage larval brains contain over 1200 cells based on nuclear counts (likely an underestimate). There are 10 neurons in the brain with 5HT-LIR, and the cell bodies are similarly positioned as at stage 9, dorsally and posteriorly (Fig. 8a; Additional file 6c). Similar to stage 9, many neurons in the juvenile brain have FMRF-LIR (Fig. 9a, b, d). Finally, *Ct-syt1* is expressed throughout the juvenile brain (Fig. 10a, c, d), with the exception of the central neuropil (np). This expression pattern is very similar to that seen in stage 9 larvae (*e.g.,* Fig. 7o). Although the overall morphology of the brain does not dramatically change during metamorphosis, we do detect some small differences between stage 9 larvae and juveniles. For example, we counted only 10 neurons with 5HT-LIR in the brains of 7-day old juveniles versus 10 to 14 neurons with 5HT-LIR at stage 9. Another difference is that there is no trace of the sc^ac+^ in 7-day old juveniles (Fig. 8a, a’).

Another difference between the nervous system of stage 9 larvae and 7-day old juveniles is the loss of neurites with aTUB-LIR and 5HT-LIR underlying the position of the larval prototroch and telotroch. This is not surprising since the trochal bands are no longer present after metamorphosis (Fig. 8; Additional file 6a, b). Another difference is that several neurons with FMRF-LIR that were visible at late-larval stages are no longer visible seven days after metamorphosis, including those in the dorsal-anterior epidermis (data not shown) and F-AMC (Fig. 9e, e’). Finally, there are several new neurons with FMRF-LIR in the ventral head epidermis (compare Fig. 9e, e’ with Fig. 6f, f’).

Within the ventral nerve cord, the size and shape of the first thirteen ganglia are similar to that seen at stage 9. However, the ganglia are spaced farther apart in juveniles (compare Fig. 8a, b with 5e, g), likely due to the overall body elongation that occurs as a result of metamorphosis. Moreover, juveniles continue to add new segments, including new ganglia, from a posterior growth zone after metamorphosis [43], so the number of ganglia in the ventral nerve cord varies from animal to animal. For example, all of the juveniles imaged in this study were seven days old, but the animal in Fig. 8b has 14 ganglia (versus the 13 found in stage 9 larvae) plus one newly-forming ganglion at the posterior end while the animal in Additional file 7a has 17 ganglia plus one newly-forming, posterior ganglion. All of the thoracic and abdominal ganglia have subsets of neurons with 5HT-LIR (Fig. 8) and with FMRF-LIR (Fig. 9e, e’; Additional file 7a). It is difficult to discern a bilaterally-symmetric pattern of each neural subtype (5HT-LIR and FMRF-LIR) between hemiganglia in the same segment, although there appear to be similar numbers of each type of neuron on each side. The lack of bilateral symmetry between neural subtypes in the ventral nerve cord was reproducible across multiple animals and experiments. In contrast, clear bilateral symmetry was consistently visible for other neural elements such as peripheral nerves, connectives in the ventral nerve cord, and neuronal soma with 5HT-LIR and FMRF-LIR in the brain. Finally, the soma within each ganglion of the ventral nerve cord express *Ct-syt1* (Fig. 10a, b) as they did at stage 9. As new segments are added, cells within the newly forming ganglion also express *Ct-syt1* (Fig. 10a, b open arrow).

When comparing the thoracic versus abdominal portion of the ventral nerve cord, similar regional differences as those found at stage 9 can be seen. For example, there is a clear difference between thoracic and abdominal segments in the size and shape of ganglia and the pattern of peripheral nerves (Fig. 8b compare bracketed thoracic segment 3 with abdominal segment 11; Fig. 10a, b). The thoracic portion of the ventral nerve cord has large ganglia, two paired main peripheral nerves per segment (one medial and one posterior), and left and right connectives that are closely juxtaposed (Fig. 8a, a’, B, b’; Additional file 6f – f”’). The first and 9th thoracic ganglia are also somewhat different, the first being much larger than the other thoracic ganglia and the ninth being much smaller and more similar in appearance to abdominal ganglia (Fig. 8; Fig. 10a, b).The abdominal portion of the ventral nerve cord has smaller ganglia, three pairs of peripheral nerves per segment, connectives that are farther apart, and a clearly visible medial connective (Fig. 8; Additional file 6g – g”’). Both the thoracic and abdominal ganglia abut the posterior edge of each segment (segments 3 and 11 are bracketed in Fig. 8b; the posterior boundary of segments 4 and 11 is indicated with lines in Additional file 7a). Within one abdominal segment from anterior to posterior, the first peripheral nerve after the anterior segment boundary is a thin nerve (minor peripheral nerve, Fig. 8b’ open arrowhead points to one). Then there is the anterior boundary of the ganglion, followed by a thick nerve (main peripheral nerve, Fig. 8b’ asterisk marks one) in the middle of the ganglion. The third peripheral nerve (main peripheral nerve, Fig. 8b’ filled circle marks one) is at the posterior edge of the ganglion, which abuts the posterior edge of the segment.

Most elements of the stomatogastric nervous system visible at stage 9 are also present in juveniles. The pair of ganglia lateral to the posterior edge of the dorsal pad of the pharynx is still visible and expresses *Ct-syt1* (Fig. 10e open arrowheads). These *Ct-syt1*^+^ ganglia coincide in location with cells that have FMRF-LIR (Fig. 9c, d’; Additional file 7a open arrowheads). A single cell with FMRF-LIR is visible on the dorsal-anterior side of the dorsal pad of the pharynx (Fig. 9c, d’ closed arrowhead). The ganglia on either side of the dorsal pad of the pharynx extend nerves anteriorly (anterior enteric nerves, aENs) and ventrally (ventral-posterior enteric nerves, vpENs). The pair of anterior enteric nerves (aENs) runs along each dorsal-lateral side of the dorsal pad, which is visible by FMRF-LIR (Fig. 9c, d, d’) and aTUB-LIR (Fig. 8a’; Additional file 6d). Each aEN branches (Fig. 8a’; Fig. 9c, d’; Additional file 6d open arrow) and projects medially along the anterior side of the dorsal pad (Fig. 9d, Additional file 6d) and ventrally towards the mouth (Fig. 8a’; Fig. 9c). The ventral branch of each aEN has additional branch points: one thick branch (posterior to the position of the mouth) extends ventrally and posteriorly towards the ventral nerve cord, two thin branches (posterior to the position of the mouth) extend posteriorly and medially towards the ventral side of the pharynx, and one thick branch (anterior to the mouth) initially extends ventrally and then turns dorsally and joins the circumesophageal connective just before it enters the brain (Fig. 8a’; Fig. 9c and data not shown). It appears that only a subset of each aEN nerve has FMRF-LIR, and it is not clear if all of the branches have FMRF-LIR. Each ventral-posterior enteric nerve (Fig. 8a’ vpEN) is thinner than the aENs and initially projects ventrally before branching. One branch extends anteriorly and runs along the ventral pharynx and the other branch extends posteriorly towards the esophagus.

Additional elements of the stomatogastric nervous system include thin neurites with FMRF-LIR that extend from the two ganglia and from the aENs into the lateral sides of the pharynx and posteriorly towards the esophagus (data not shown). Additional, thin neurites with aTUB-LIR and 5HT-LIR innervate the dorsal pad (Additional file 6d, e). Furthermore, cell bodies and neurites with FMRF-LIR are present along the length of the juvenile esophagus (Additional file 7b open arrows), midgut and hindgut (Additional file 7b and data not shown).

Other features visible in juveniles by aTUB-LIR include the nuchal organs (Fig. 8a’, b’ closed arrowheads), the metanephridia (Fig. 8b’ open arrows), and tufts of cilia all over the head and on the lateral and dorsal sides of the body, and in the hindgut (Fig. 8a, b; Additional file 6a). The nuchal organs extend neurites into the brain (data not shown) as do the tufts of cilia on the prostomium (data not shown). Neurites with aTUB-LIR and 5HT-LIR can also be seen extending throughout the epidermis of the trunk (Fig. 8a’, a”, b’, b”; Additional file 6a, b). Overall, the nervous system in juveniles is very similar to that seen in stage 9 larvae, suggesting that the mature larval nervous system and the juvenile nervous system are one in the same in *C. teleta*.

## Discussion

Annelids exhibit a wide range of life histories, including varying degrees of direct versus indirect development and planktotrophy versus lecithotrophy. *Capitella teleta* has an indirectly-developing, lecithotrophic, non-feeding larval phase, during which time most of the tissues being formed appear to be those of the juvenile with very few “larval-specific” tissues. Accordingly, few cells are lost or dramatically rearranged during metamorphosis. For example, the brain and ventral nerve cord are very similar in appearance between stage 9 and one-week old juveniles (*e.g.,* compare Fig. 8a, b with Fig. 5e, g). This mode of development is comparable to what has been described for several mollusks. It has been proposed that the molluscan larval body largely consists of forming juvenile structures, but with the presence of a few transient structures (*e.g.,* prototroch and apical sensory organ) to allow for a planktonic phase [9].

*Comparison of adult nervous system formation in* C. teleta *with other annelids*. The nervous system of *C. teleta* shares many features with other annelids such as the morphological arrangement of nerves and the presence of widespread 5HT-LIR and FMRF-LIR throughout the central nervous system and stomatogastric nervous system [11, 13, 36]. It has been proposed that the ancestral state for the anatomical organization of the central nervous system in annelids is four cerebral commissures, paired circumesophageal connectives, an unknown number of segmental peripheral nerves, and a ventral nerve cord with multiple commissures and five longitudinal connectives that later fuse into three [11, 48]. The ancestral state in the number and arrangement of segmental nerves and commissures in annelids is still unclear, in part because fusion of bundles of neurites in adults makes cross-species comparisons more difficult. There are a wide variety of organizational patterns of central nervous systems in adults of different annelid taxa [11, 48].

**Fig. 11:**
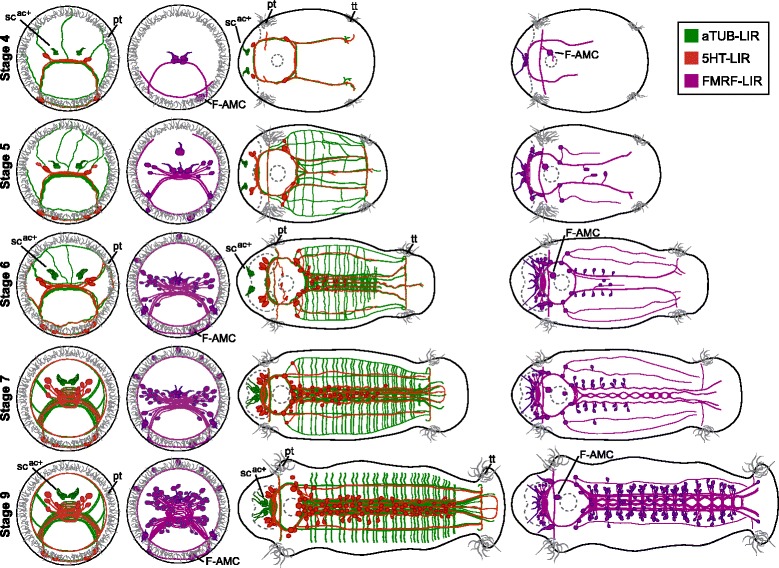
Diagrams of 5HT-LIR, aTUB-LIR and FMRF-LIR in *C. teleta* larvae. aTUB-LIR is shown in green, 5HT-LIR in red and FMRF-LIR in purple. The stomatogastric nervous system is omitted for clarity. The left two columns are anterior views with ventral down. The right two columns are ventral views with anterior to the left. Stage is indicated to the left of each row. F-AMC, FMRF-LIR anterior mouth cell; pt, prototroch; sc^ac+^, acetylated tubulin-positive sensory cells; tt, telotroch

In comparison with other annelids, the brain of *C. teleta* is relatively simple in its morphological organization. There are two brain lobes joined by a commissure and connected to the ventral nerve cord by circumesophageal connectives. In larvae and juveniles of *C. teleta*, we did not identify clear subdivisions of the brain neuropil into dorsal and ventral commissures nor did we see paired circumesophageal connectives on the left and right sides (Fig. 11), although we expect that examination of brain architecture in mature *C. teleta* adults and use of a wider set of neural markers will reveal additional structures and subdivisions. We did not identify any morphological subdivisions along the anterior-posterior axis in the brain or any discrete ganglia along the circumesophageal connectives. The simple organization of the brain may reflect a deposit-feeding lifestyle, which is accompanied by a reduction in parapodia and sensory structures as is seen in other taxa within Sedentaria [2, 5, 51].

The ventral nerve cord in *C. teleta* has many features characteristic of a ladder-like nerve cord, with segmentally-iterated ganglia, longitudinal connectives between ganglia in adjacent segments, and commissures between each hemiganglion within one segment. Initially the ventral nerve cord in *C. teleta* has 5 longitudinal connectives (Fig. 11). These appear to fuse into two main bundles in the juvenile, although in the abdominal segments, a thin medial connective (aTUB-LIR) and four outer connectives (5HT-LIR) can still be seen. Thus, *C. teleta* has the ancestral pattern of five connectives. The exact number of commissures within each ganglion is difficult to discern. There are two pairs of peripheral nerves in the thoracic segments and three pairs of peripheral nerves in the abdominal segments. The numbers of nerves in *C. teleta* fall within the range seen in other annelids, and this study highlights the advantage of characterizing nervous system architecture during development since the number and arrangement of segmental nerves is often easier to determine. It will be interesting to compare ventral nerve cord development across annelids as more data become available to see if a more obvious ancestral pattern of neural architecture emerges.

Innervation of the stomatogastric system in annelids is less well-described using immunohistochemical or molecular techniques, and an ancestral pattern has not been proposed [11, 48]. In late-stage larvae and one-week old juveniles of *C. teleta*, we see two ganglia on either side of the dorsal pad of the pharynx that express *Ct-syt1* (*e.g.,* Fig. 10e open arrowheads) and have FMRF-LIR. These ganglia were previously described for *Capitella capitata* by Eisig [52]. The pattern of stomatogastric nerves found in *C. teleta* is very similar to available descriptions from other annelids [4, 28, 35, 36], with two pairs of nerves that join the circumesophageal connectives and brain neuropil, and a pharyngeal nerve ring. There are several additional stomatogastric nerve branches (described above in the juvenile results section), and a small subset of stomatogastric neurites that have 5HT-LIR and aTUB-LIR, which is visible as a plexus in the dorsal pad of the pharynx. Innervation of the intestine and hindgut is visible by aTUB-LIR and FMRF-LIR, and cell bodies with FMRF-LIR can be seen along the entire length of the intestine. One feature that is shared among several annelid species is innervation of the foregut by two pairs of nerves, one pair arising from the brain neuropil and one pair arising from the circumesophageal connectives. These nerves form a ring around the outside of the pharynx in some species and contribute to a plexus innervating the walls of the proboscis [4]. Recent studies have identified neurites and nerves with 5HT-LIR and FMRF-LIR around the pharynx of *Pomatoceros lamarckii*, *Sabellaria alveolata* and *Polygordius lacteus* larvae and along the juvenile intestine in *P. lamarckii* [28, 35, 36]. Interestingly, neither 5HT-LIR nor FMRF-LIR was found in the developing stomatogastric nervous system of *Hydroides elegans* larvae. Instead, cells with tyrosine hydroxylase immunoreactivity were associated with the pharynx [33]. Taken together, a large component of the stomatogastric nervous system in *C. teleta* has FMRF-LIR and the overall pattern of innervation is similar to descriptions for several other annelids. Furthermore, we provide clear evidence of two stomatogastric ganglia on the left and right sides of the pharynx in *C. teleta*.

*Elements of the larval nervous system in* C. teleta. Although *C. teleta* largely forms its juvenile nervous system during larval development, there are a few transient larval structures present during this time. These include ciliary bands used for locomotion (prototroch and telotroch), ciliated cells of the neurotroch and pygidium, and the larval photoreceptor cells [55, 56]. Innervation of the prototroch and telotroch is evident by 5HT-LIR and FMRF-LIR (Fig. 11), and this innervation is no longer visible after metamorphosis (Fig. 8, Additional file 7). Several other annelid larvae have been reported to have both 5HT-LIR and FMRF-LIR in their prototrochal nerve ring [13, 33, 35, 36], while others have been reported to have only 5HT-LIR [28, 37]. Furthermore, 5HT-LIR in the prototrochal ring is common among spiralian larvae [10]. It remains to be seen if this pattern of prototrochal innervation is well conserved since only a few neurotransmitter subtypes and their synaptic targets (*e.g.,* muscle cell versus ciliary band cell) have been examined in more than one species of annelid [57]. In *C. teleta*, innervation of the neurotroch is more difficult to discern; thin projections underlying the ciliated cells are visible by aTUB-LIR, but it is not clear if these are neurites or extensions from the ciliated cells themselves. After metamorphosis, neither the neurotroch nor the aTUB-LIR underlying the neurotroch is visible. In summary, the trochal bands and the neurites underlying them are lost after metamorphosis and constitute a larval feature.

Another transient larval character typical of spiralians is the apical sensory organ and associated apical tuft. The spiralian apical sensory organ is hypothesized to play a role in sensing metamorphic cues [58, 59], and ablation of the apical sensory organ in the gastropod mollusk *Phestilla sibogae* prevents induction of metamorphosis [60]. Where examined, the spiralian apical sensory organ consists of multiple cell types (flask-shaped cells with neurites that extend to the surface and ciliated cells) that exhibit reactivity for a variety of cross-reactive antibodies (serotonin, FMRFamide, catacholamines, leu-enkephalin and tubulin) [6, 8, 13, 28, 33, 61, 62]. *Capitella teleta* larvae do not have a clearly demarcated apical sensory organ or an apical tuft, but they do delay metamorphosis until an appropriate cue is present [63]. *Capitella teleta* possess bilateral pairs of cells with strong aTUB-LIR (sc^ac+^) that develop early in the anterior ectoderm and send projections to the surface (Fig. 11). These cells eventually come to be positioned in the dorsal-medial, anterior region of the brain and are absent after metamorphosis. The sc^ac+^ display some morphological similarity to cells in the apical sensory organ of gastropod veliger larvae. For example, the EA cells in *Ilyanassa obsoleta* are vase-shaped, send dendrites to the surface of the head, and are strongly labeled with an anti-α-tubulin antibody [8]. Kempf *et al.* (2005) described another group of cells with similar properties, the ampullary cells, as having “thick, dense bundles of internalized, modified cilia” in seven different species of opistobranchs [61]. However, in the larva of the annelid *P. dumerilli,* flask-shaped cells with high levels of anti-acetylated tubulin staining were found outside the apical sensory organ in the head and were identified as cells in the akrotroch (act, [29]). *Capitella teleta* has additional flask-shaped cells with FMRF-LIR that are positioned in the developing brain and send projections to the anterior surface (Fig. 11). It is possible that in *C. teleta* some of these flask-shaped cells (cells with FMRF-LIR or sc^ac+^) are remnants of the apical sensory organ and have come to be positioned within the brain. It has been suggested that a chemosensory pyridine receptor is responsible for triggering metamorphosis in *C. teleta* [64], possibly via release of serotonin [65, 66]. It remains to be seen which cells in *C. teleta* are responsible for sensing the metamorphic cue, and we did not detect any flask-shaped cells with 5HT-LIR, which would have been good candidates.

*C. teleta* shares additional larval nervous system features with other annelids. For example, the asymmetric F-AMC cell (FMRF-LIR anterior mouth cell; Fig. 11) is very similar in appearance to an asymmetric cell with FMRF-LIR (fv1 cell) in *Phyllodoce maculata* [13]. The S-PC cell (5HT-LIR pygidial cell; Additional file 1f, closed arrowhead) in *C. teleta* is very similar in appearance to a posterior cell with 5HT-LIR in the pygidium that is found in several other developing annelids (sp1 in *Phyllodoce maculata* [13], psc in *Pomatoceros lamarkii* [28], *Platynereis dumerilli* [29, 67], S-PC in *H. elegans* [33], *Chaetopterus sp.* (NPM unpublished data). Within the annelids mentioned, the soma of this posterior serotonergic cell has a conserved ‘horn-like’ shape with a pair of neurites that extend anteriorly along the left and right lateral sides of the body. Each neurite then bifurcates and projects around the circumference of the larva at the position of the prototroch. This cell is visible by the end of gastrulation, but appears to be transient, disappearing before or during metamorphosis where examined. In *Chaetopterus sp.* and *H. elegans*, this cell protrudes from the posterior end of the larvae and is closely associated with a single, long projection that extends into the external environment, possibly a cilium. In *C. teleta*, the S-PC cell also has a horn shape and sends a pair of neurites anteriorly along the left and right sides of the larva. However, S-PC exhibits several differences from the other annelids mentioned. It does not protrude from the posterior end of the larva or have a visible cilium. In addition, S-PC was only detected after development of the brain and ventral nerve cord, which contrasts with the early appearance of this horn-shaped cell in other annelids. We cannot be certain if the bilateral neurites bifurcate at the position of the prototroch due to the late appearance of S-PC in *C. teleta* and the presence of other neurites with 5HT-LIR in this position at later stages. Neither F-AMC nor S-PC is visible after metamorphosis in *C. teleta*, suggesting that these are both larval neurons.

*Hypotheses of the ground-pattern of nervous system development in annelids*. The ancestral pattern of nervous system development in annelids is currently under debate. One hypothesis is that nervous system development begins in the anterior ectoderm. In this scenario, anterior neurons send neurites towards the posterior end of the larva, and these neurites serve as a scaffold for development of the ventral nerve cord [11, 35, 68]. Another hypothesis is that the nervous system simultaneously develops in the anterior and posterior ectoderm, and both groups of neurons send connectives towards each other to give rise to the complete central nervous system [5, 11]. Differing relationships between the developing larval and adult nervous systems have also been observed, ranging from independent formation of the two [69], incorporation of the larval into the adult nervous system [35], or formation of the adult nervous system along neurites of pioneer larval neurons [13, 27, 70–72]. It has also been hypothesized that the adult nervous system begins forming earlier during development in lecithotrophic versus planktotrophic larvae [11, 73].

*Capitella teleta* has a lecithotrophic larva in which the general pattern of nervous system development is from central to peripheral and anterior to posterior based on all of the markers examined in this study. The first neurons differentiate in the forming brain early during stage 4 as seen by *Ct-syt1* expression. We do not know what neurotransmitter is expressed in the first neurons since neurites with aTUB-LIR can be seen extending from the developing brain towards the posterior before the first neurons with 5HT-LIR or FMRF-LIR are visible. Although these neurons are clearly forming within the central nervous system, we cannot be certain if they are larval or adult neurons (discussed below). Shortly after appearance of the *Ct-syt1*^+^ cells in the brain, additional neurons with 5HT-LIR and FMRF-LIR in the brain and a larval neuron with FMRF-LIR on the left-anterior side of the mouth (F-AMC) become visible. By the end of stage 4 and the beginning of stage 5, neurons begin to differentiate around the mouth and by the end of stage 5 and beginning of stage 6, neurons start differentiating in the forming ventral nerve cord (Fig. 11). Increasing numbers of ganglia with differentiating neurons (*Ct-syt1*^+^ cells) can be seen from the beginning to the end of stage 6 (compare Fig. 4h with k), presumably due to the progressive differentiation of neurons from anterior to posterior.

Initiation of development of the anterior central nervous system, which largely contributes to the juvenile nervous system, prior to clearly-identified larval neurons in *C. teleta* would seem to support the hypothesis that the juvenile/adult nervous system forms earlier than the larval nervous system in lecithotrophic larvae. However, the situation is complex in *C. teleta,* and larval neurons can be found in both the periphery and the central nervous system. For example, there are neurons with 5HT-LIR in the brain that send neurites to the prototroch. The neurites underlying the prototroch are lost after metamorphosis, suggesting that these neurons serve a larval function. However, it is not clear what happens to their soma because, although there is a reduction in the number of neurons with 5HT-LIR in the brain (from 14 to 10), this loss can occur two days prior to metamorphosis. This implies that these neurons are retained and serve another function after metamorphosis. Although *C. teleta* does not have an apical organ, another example is the presence of flask-shaped cells with FMRF-LIR and aTUB-LIR in the brain. One clear larval neuron is F-AMC, which is located on the left side of the mouth and is lost after metamorphosis. F-AMC is the earliest larval neuron detected in this study, but it forms after neurons in the brain are present.

In *C. teleta*, the first neurons form in the brain and send axons posteriorly along the edges of the ventral neural ectoderm (*e.g.,* Fig. 2a, b). The position of these neurites in *C. teleta* changes as development proceeds, following the edges of the converging and extending ventral neural ectoderm (Fig. 2a – c). Interestingly, we frequently observed aberrant neurites in the trunk that were positioned outside of the region of the ventral nerve cord during early stages of nervous system development (*e.g.,* Fig. 2f”; Additional file 2d’, d’ open arrow), but are absent in late-stage larvae. These aberrant neurites did not appear to result in formation of ectopic ganglia. Instead, aberrant neurites may be pruned as part of a normal refinement process. Experimental ablation of the early neurons in the brain would determine whether their neurites are necessary for initiation of ventral nerve cord development, or if their outgrowth is a response to cues previously established by the ventral neural precursor cells themselves. In conclusion, neural development in *C. teleta* progresses from anterior to posterior and from central to peripheral. This pattern of neural development is different from the early formation of peripheral, larval neurons at various locations throughout the body seen in some spiralian taxa with planktotrophic larvae (*e.g.,* [13, 28, 32, 33]). The mode of neural development in *C. teleta* may be due to the paucity of larval structures or to lecithotrophy, and additional data that unambiguously identifies whether or not the first neurons in the brain contribute to the juvenile nervous system will be important in addressing this question.

Species-specific differences in where the first neurons differentiate and the relative timing of larval versus adult nervous system formation may be explained by variation in life histories. Lecithotrophic larvae have maternally-deposited food stores that they can immediately use to start building their juvenile or adult body, including the juvenile or adult nervous system. Adult nervous system formation often begins with the brain, which is located in the anterior. In contrast, planktotrophic larvae require larval neurons to be functional early in development for feeding and associated behaviors, and these neurons can form throughout the body. In this scenario, the anterior adult nervous system would form earlier in lecithotrophic larvae versus disparately-localized larval neurons in planktotrophic larvae [11, 73–76]. However, patterns of larval versus adult nervous system formation vary across annelid species, and available examples do not easily fit this dichotomous pattern.

The following three taxa all have planktotrophic larvae, but vary in the type (larval versus adult) and location of the first neural elements to appear. In the planktotrophic larva of *Pomatoceros lamarckii*, the first neurons detected were cells with FMRF-LIR in the apical organ of the larva and a posterior, peripheral cell with 5HT-LIR (psc) [28]. In general, the apical organ is considered to be a larval structure, so in this species larval neurons form first in both the anterior and posterior. In the planktotrophic larva of *Phyllodoce maculata*, the first neuron detected was a posterior larval neuron with 5HT-LIR (sp1) followed several hours later by an anterior neuron with FMRF-LIR in the apical organ (fa1) [13]. In this species, larval neurons form first in the posterior and then in the anterior. In contrast to both of these examples, in the planktotrophic larva of *Polygordius lacteus*, the first neurons detected were cells with 5HT-LIR and FMRF-LIR in the apical ganglion and cells with 5HT-LIR in the subesophageal ganglion, all of which later contribute to the adult nervous system [35]. This study did not look at earlier stages of development to determine which neurons form first; however, as both sets of neurons detected are part of the central nervous system and no peripheral neurons were identified at this time, it is likely that central elements form first in this species. This variability highlights the need for data from more annelid taxa with different life histories, and the use of pan-neuronal markers. It will also be important to clearly identify larval versus adult components of the nervous system in order to understand how neural development relates to different life-history strategies.

Data from additional spiralian taxa will be instrumental in addressing the broader question of how nervous system development relates to life history strategies. For example, the polyclad flatworm *Maritigrella crozieri* has a planktotrophic larva, and the first neuron observed with 5HT-LIR forms in the anterior periphery near the epidermal eye shortly after completion of gastrulation. Following this, a pair of neurons with 5HT-LIR forms in the developing brain and one with 5HT-LIR forms in the posterior periphery [32]. These data support the hypothesis that in planktotrophic larvae, peripheral larval neurons differentiate before the central nervous system begins forming. In contrast, the brachiopod *Novocrania anomala* has a lecithotrophic larva, but the first neurons with 5HT-LIR are only visible once the larvae are competent to metamorphose, very late in larval development. Furthermore, these neurons are flask-shaped cells in the anterior-most part of the apical lobe and are no longer visible after metamorphosis, suggesting that they are larval-specific neurons [77]. It is not clear when the central nervous system begins forming in *N. anomala*, so it is difficult to determine if the central nervous system forms before or after the first peripheral larval neurons form. There are additional data available on nervous system development using neuronal subtype markers from brachiopod species with either planktotrophic or lecithotrophic larvae, for example see [78, 79], but the authors do not discuss where the earliest neurons differentiate in each of these studies. A homolog of *synaptotagmin* 1 (*Tt-synaptotagmin* 1) in the lecithotrophic larva of the brachiopod *Terebratalia transversa* is expressed early during gastrulation in a small area of animal cap ectoderm [80]. Expression in the anterior ectoderm is maintained as additional regions of expression in the apical lobe are added. These results suggest that the first neurons in *T. transversa* may differentiate in the anterior ectoderm, a region that contains both larval neurons of the apical organ and neurons in the central nervous system. The addition of more taxa and the use of pan-neuronal markers for cross-species comparisons will be important for elucidating the relationship between life-history characteristics and neural development.

## Conclusions

*C. teleta* appears to largely form its juvenile nervous system during the larval phase, although some larval-specific neurons such as F-AMC are present early and may be homologous with larval neurons in other annelid species. The first neurons to form are those in the brain, and development of both neurons and neurites proceeds from central to peripheral and from anterior to posterior in *C. teleta. Capitella teleta* shares many features of its nervous system with other annelids, including an anterior brain, circumesophageal connectives, a ladder-like ventral nerve cord with reiterated commissures and five main connectives and multiple reiterated peripheral nerves. Some of these shared features such as the five connectives in the ventral nerve cord are only visible during larval stages, highlighting the importance of including developmental data in ancestral character state reconstructions. Although we do not yet have enough data to reconstruct the ancestral pattern of annelid nervous system development, continued efforts towards this goal will be crucial toward enhancing our understanding of body plan evolution.

## Methods

### Animal care

*Capitella teleta* adults [26] were maintained in the lab as described in [43] with the following exceptions. Animals were kept in either 20 μm filtered seawater (FSW) or artificial seawater (ASW) at 19 °C. ASW was made by mixing 2 cups (~559.8 g) of Instant Ocean Sea Salt into 15.14 L distilled water. This solution was vigorously mixed and then allowed to sit overnight. Salinity was adjusted the following day to a specific gravity of 1.023 – 1.025 at 22 °C, which was measured with a hydrometer.

### Immunohistochemistry

Depending on age, animals were treated for fixation as follows:

1) Stages 4 – 9 were incubated at room temperature (r.t.) in a 1:1 mixture of 0.37 M MgCl_2_ and FSW or ASW for 5 – 15 minutes, and then fixed for 15 – 30 minutes at room temperature with 4 % paraformaldehyde (diluted from 32 % paraformaldehyde ampules from Electron Microscopy Sciences) in FSW or ASW. Following fixation, the animals were washed several times with phosphatase buffered saline (PBS) and PBS + 0.1 % Triton X-100 (PBT) and then stored in PBS at 4 °C.

2) 7-day juveniles were extracted from mud into a 0.6 % corn meal agar (Sigma) (CMA) plate in order to allow food to be cleared from the gut. Juveniles were then transferred to a 0.6 % CMA plate with MgCl_2_ (0.6 g corn meal agar in 50 mL FSW and 50 mL 0.37 M MgCl_2_) for twenty minutes at r.t. for relaxation. Juveniles were then fixed in 4 % paraformaldehyde in FSW for 1 hour at r.t. Following fixation, animals were washed 4 times with PBT and stored in PBS at 4 °C.

Following fixation, all animals were incubated in block consisting of 10 % heat-inactivated normal goat serum (Sigma and Life Technologies) in PBT for 1 hour at r.t. while rocking. Animals were incubated in primary antibodies in block overnight at 4 °C while rocking. Following 2 quick washes with PBT, animals were washed 4 times for 30-minute intervals while rocking before incubating with secondary antibodies in block for a few hours at r.t. or overnight at 4 °C. Animals were quickly rinsed twice with PBT before washing 7 times for 10-minute intervals at r.t. while rocking. Animals were then either washed in PBS and incubated in 1:1000 TO-PRO-3 iodide in PBS (Life Technologies) or incubated in 80 % glycerol in PBS with 0.1 μg/mL Hoechst at 4 °C while shaking for two or more days (up to 3 months). Animals that had been incubated in TO-PRO-3 were washed several times quickly in PBS over 30 minutes at r.t. and then cleared in SlowFade Gold or ProLong Gold mounting medium (Life Technologies).

Primary antibodies are as follows: 1:1600 rabbit anti-FMRFamide (Immunostar, cat #20091), 1:600 – 1:800 mouse anti-acetylated-α-tubulin (clone 6-11B-1, Sigma), 1:400 rabbit anti-5HT (Immunostar, cat #20080), 1:800 rabbit anti-5HT (Sigma, cat #S5545) and 1:500 mouse anti-histone (clone F152.C25.WJJ, Millipore). Secondary antibodies are as follows: 1:400 goat anti-mouse FITC, 1:800 goat anti-mouse AlexaFluor 488, 1:500 donkey anti-mouse AlexaFluor 647, 1:400 – 1:600 donkey anti-rabbit AlexaFluor 546, 1:600 donkey anti-rabbit AlexaFluor 647, and 1:400 anti-rabbit Rhodamine from Life Technologies and 1:1000 sheep anti-rabbit F(ab′)_2_ Cy3 from Sigma.

### Cloning *Ct-synaptotagmin1*

Total RNA was collected from mixed stage 1 – 9 *C. teleta* embryos and larvae. 5'- and 3'- RACE ready cDNA was synthesized using the SMARTer RACE kit (Clontech). A fragment of the *Ct-synaptotagmin* 1 homolog was identified by BLAST analysis of the *C. teleta* genome (JGI, DOE) and amplified by PCR using gene-specific primers. The resulting 641-bp fragment, spanning part of the C2A domain and the entire C2B domain, was ligated into the PGEM T-Easy vector (Promega) and sequenced. This sequence has been deposited in GenBank as *Ct-synaptotagmin 1*, accession number KR063024.

### Whole mount in situ hybridization (WMISH)

Stage 4 – 9 larvae and juveniles were treated for fixation as described above. Stage 3 embryos were treated for 3 minutes in a 1:1 solution of 1 M sucrose and 0.25 M sodium citrate, washed 3 times in FSW or ASW and then fixed. All fixations for WMISH were carried out in 4 % paraformaldehyde in FSW or ASW for 6 hours – overnight at 4 °C. After fixation, animals were dehydrated in methanol and stored at −20 °C. WMISH was carried out as previously described in [81, 82]. Briefly, animals were hybridized for ~72 hours at 65 °C with 1 ng/μL of DIG-labeled anti-sense *Ct-syt1* RNA probe that was generated using the MegaScript T7 transcription kit (Life Technologies). The color reaction was carried out using 330 μg/mL of nitroblue tetrazolium and 165 μg/mL 5-bromo-4-chloro-3-indolyl phosphate in alkaline phosphatase buffer.

### Imaging

WMISH images were taken using DIC optics on a Zeiss M2 compound microscope coupled with an 18.0 megapixel EOS Rebel T2i digital camera (Canon). All animals labeled by immunohistochemistry were imaged by confocal laser scanning microscopy. Animals labeled with anti-FMRF were imaged using an LSM 710 (Zeiss). Stage 4 – 6 larvae and juveniles labeled with anti-serotonin and anti-acetylated-α-tubulin were imaged using an LSM 510 (Zeiss). Stage 7 – 9 larvae labeled with anti-serotonin and anti-acetylated-α-tubulin were imaged using a TCS SP5-X (Leica). Z-stack projections were generated using ImageJ (NIH). Some WMISH images were joined into a composite image using Helicon Focus 6.2.0 (Helicon Soft). Images (confocal and WMISH) were edited using Photoshop CS4 and figure panels were constructed using Illustrator CS4 (Adobe Systems Inc.).

## Additional files

Additional file 1:
**aTUB-LIR and 5HT-LIR in early-stage** ***C. teleta*** **larvae (stages 4 – 6).** Images are z-stack confocal images of larvae labeled with anti-acetylated-α-tubulin (green), anti-serotonin (red) and anti-histone (blue). Panels labeled with an apostrophe (*e.g.,* A') are single-channel images of either aTUB-LIR (’) or 5HT-LIR (”) from the merged image without an apostrophe (*e.g.,* A). All panels are to the same scale unless otherwise noted. G is a cropped, 2.7× magnified view of the pygidium. The position of the prototroch and telotroch is indicated in A, B, C, E and F, and the position of the telotroch is indicated in G. The closed arrowhead in A” points to the left soma with 5HT-LIR in the brain. The closed arrows in C’ point to the left longitudinal neurites in the dorsal-lateral and ventral-lateral trunk. The open arrows in C” point to neurites with 5HT-LIR that run along the prototroch and telotroch. The closed arrowhead in C” and G” points to S-PC. The open arrow in D points to the first neurites with aTUB-LIR. The open arrowhead in E and F marks the sc^ac+^. The closed arrowhead in F points to a posterior cell body with 5HT-LIR. Stage is indicated in the lower-left corner, and view is indicated in the lower-right corner of the merged panels. All lateral views are of the left side. Anterior is to the left in all lateral views, and ventral is down in all anterior views. ant, anterior; lat, lateral; pt, prototroch; tt, telotroch. Additional file 2:
**Progression of the ventral nerve cord from stage 4 – 6 (aTUB-LIR and 5HT-LIR).** Images are z-stack confocal images of larvae labeled with anti-acetylated-α-tubulin (green), anti-serotonin (red) and nuclear stain (blue). Panels labeled with an apostrophe (*e.g.,* A') are single-channel images of either aTUB-LIR (’) or 5HT-LIR (”) from the merged image without an apostrophe (*e.g.,* A). The position of the prototroch and telotroch is indicated in A – G. The brain neuropil (np), circumesophageal connectives (cc), subesophageal commissure (sc) and main connectives (mc) of the ventral nerve cord are indicated in A’. The open arrowheads in C’ point to forming commissures, and the closed arrowhead in C” indicates the position of the ventromedian connective. The open arrows in D’ and D” point to an aberrant neurite, while the open arrowheads in D’ point to the paramedian connectives. Panels E – G show the lengthening and narrowing of the neurotroch (nt); the prototroch (pt) and telotroch (tt) are also indicated. All are ventral views with anterior to the left. Stage is indicated in the lower-left corner. cc, circumesophageal connectives; mc, main connectives; np, neuropil; pt, prototroch; sc; subesophageal commissure; tt, telotroch; vent, ventral. Additional file 3:
**FMRF-LIR in early-stage** ***C. teleta*** **larvae (stages 4 – 6).** Images are z-stack confocal images of larvae labeled with anti-FMRF (red) and Hoechst nuclear stain (green). Panels labeled with an apostrophe (*e.g.,* A') are single-channel images of FMRF-LIR from the merged image without an apostrophe (*e.g.,* A). All panels are to the same scale unless otherwise noted. Panel D is a cropped, 2.2× magnified image of the head. The position of the prototroch and telotroch is indicated in A – C, E and F, and the position of the prototroch is indicated in D. The open arrowhead in A’ points to a dorsal-medial cell with FMRF-LIR. F-DMC is marked by a closed arrowhead in A’, D’, E’, F’. The first visible cells with FMRF-LIR in the ventral nerve cord are indicated with an open arrow in A’ and the lower open arrowhead in B’ and C’. In B’, a closed arrow points to a ventral-anterior flask-shaped cell with FMRF-LIR in the head, and a closed arrowhead marks a posterior cell near the ventral telotroch. Dorsal-lateral cells with FMRF-LIR are marked in B’ with the upper open arrowhead and in D’, E’, F’ with open arrowheads. Neurites that form a circumferential ring underlying the prototroch (B’, C’, E’, F’), in the mid-anterior dorsal trunk (C’, F’) and underlying the telotroch (B’, C’, F’) are labeled by the anterior, middle and posterior upper open arrows, respectively. Longitudinal dorsal trunk neurites are marked with closed arrows in C’, E’, F’. Stage is indicated in the lower-left corner, and view is indicated in the lower-right corner. All lateral views are of the left side. Anterior is to the left in all views. dors, dorsal; lat, lateral; pt, prototroch; tt, telotroch. Additional file 4:
**aTUB-LIR and 5HT-LIR in late-stage** ***C. teleta*** **larvae (stages 7 – 9).** Images are z-stack confocal images of larvae labeled with 1) anti-acetylated-α-tubulin (green) and anti-serotonin (red; A, B), 2) anti-α-acetylated-tubulin (green), anti-serotonin (red) and TO-PRO-3 nuclear stain (blue; C, D, F, G), or 3) anti-acetylated-α-tubulin (E). Panels labeled with an apostrophe (*e.g.,* A') are single-channel images of either aTUB-LIR (’) or 5HT-LIR (”) from the merged image without an apostrophe (*e.g.,* A). All panels are to the same scale unless otherwise noted. C and D are cropped, 2.27× magnified images of the head at stage 9. F is a cropped, 2× magnified image of the thoracic ganglia (1 – 9), and G is a cropped, 2× magnified view of the abdominal ganglia (10 – 13). The position of the prototroch and telotroch is indicated in A, B and E, and the position of the prototroch is indicated in C and D. In B, the bracket marks the paired main peripheral nerves, and the asterisk marks the minor peripheral nerve in segment 11. In C, the dorsal pad of the pharynx is labeled with a “px”, the open arrowheads point to the nuchal organs, and the open arrow points to the right anterior enteric nerve (aEN). The asterisk in D marks the mouth opening, and the open arrowheads point to a left pair of nerves that extend from the circumesophageal connectives towards the pharynx. In E, the brackets mark a pair of main peripheral nerves. The closed arrows in E point to dorsal-lateral longitudinal neurites with aTUB-LIR. Stage is indicated in the lower-left corner, and view is indicated in the lower-right corner. All lateral views are of the left side. Anterior is to the left in all views. lat, lateral; pt, prototroch; px, pharynx; tt, telotroch; vent, ventral. Additional file 5:
**FMRF-LIR in late-stage** ***C. teleta*** **larvae (stages 7 – 9).** Images are z-stack confocal images of larvae labeled with anti-FMRF (red) and Hoechst nuclear stain (green). Panels labeled with an apostrophe (*e.g.,* A') are the single-channel images of FMRF-LIR from the merged image without an apostrophe (*e.g.,* A). All panels are to the same scale unless otherwise noted. C and D are cropped, 1.3× magnified images of the brain. The position of the prototroch and telotroch is indicated in A, B and E, and the position of the prototroch is indicated in C and D. Open arrowheads in B’ and E’ point to two segmentally-iterated neurites that are positioned beneath the ectoderm. In D’, the closed arrowhead points to a ventral-anterior tract with FMRF-LIR in the brain neuropil, and an open arrowhead points to a dorsal-posterior tract. Closed arrows in E’ point to dorsal-lateral longitudinal neurites that are positioned beneath the ectoderm. Stage is indicated in the lower-left corner, and view is indicated in the lower-right corner. All lateral views are of the left side. Anterior is to the left in all views. lat, lateral; dors, dorsal; pt, prototroch; tt, telotroch. Additional file 6:
**aTUB-LIR and 5HT-LIR in 7-day old** ***C. teleta*** **juveniles.** Images are z-stack confocal images of 7-day old juveniles labeled with anti-acetylated-α-tubulin (green), anti-serotonin (red) and TO-PRO-3 nuclear stain (blue). A is a single-channel image showing anti-acetylated-α-tubulin and B is a single-channel image of anti-serotonin. Panels labeled with an apostrophe (*e.g.,* A') are single-channel images of either aTUB-LIR (’), 5HT-LIR (”) or TO-PRO-3 (”’) from the same z-stack as the animal in the panel without an apostrophe except for F’, which includes additional superficial focal planes to show the peripheral nerves. All panels are to the same scale unless otherwise noted. C is a cropped, 2.14× magnified image of the head; D and E are cropped, 2.14× magnified images of the pharyngeal dorsal pad; F is a cropped, 3.75× magnified images of thoracic ganglia 5 – 7; G is a cropped, 3.75× magnified image of abdominal ganglia 11 and 12. The closed arrowheads in C mark the nuchal organs. The open arrow in D points to the first branchpoint of the anterior enteric nerve (aEN). The open arrowheads in E indicate the stomatogastric ganglia. In A and B, stage is indicated in the lower-left corner, and view is indicated in the lower-right corner. In C – G, view is indicated in the lower left corner. Anterior is to the left in all views. dors, dorsal; vent, ventral. Additional file 7:
**FMRF-LIR in 7-day old** ***C. teleta*** **juveniles.** Images are z-stack confocal images of 7-day old juveniles labeled with anti-FMRF (red) and Hoechst nuclear stain (green). In A, the posterior boundaries of segments 4 and 11 are indicated with lines. In B, open arrowheads point to clusters of neurons with FMRF-LIR near the pharynx, and open arrows point to the esophagus. Stage is indicated in the lower-left corner, and view is indicated in the lower-right corner. Anterior is to the left in all views. dors, dorsal; vent, ventral.
