# Supplementary figures and images for: Nervous system development in lecithotrophic larval and juvenile stages of the annelid Capitella teleta
Source: Front Zool. 2015 Jul 11;12:15. doi: 10.1186/s12983-015-0108-y (PMC4498530; doi:10.1186/s12983-015-0108-y)

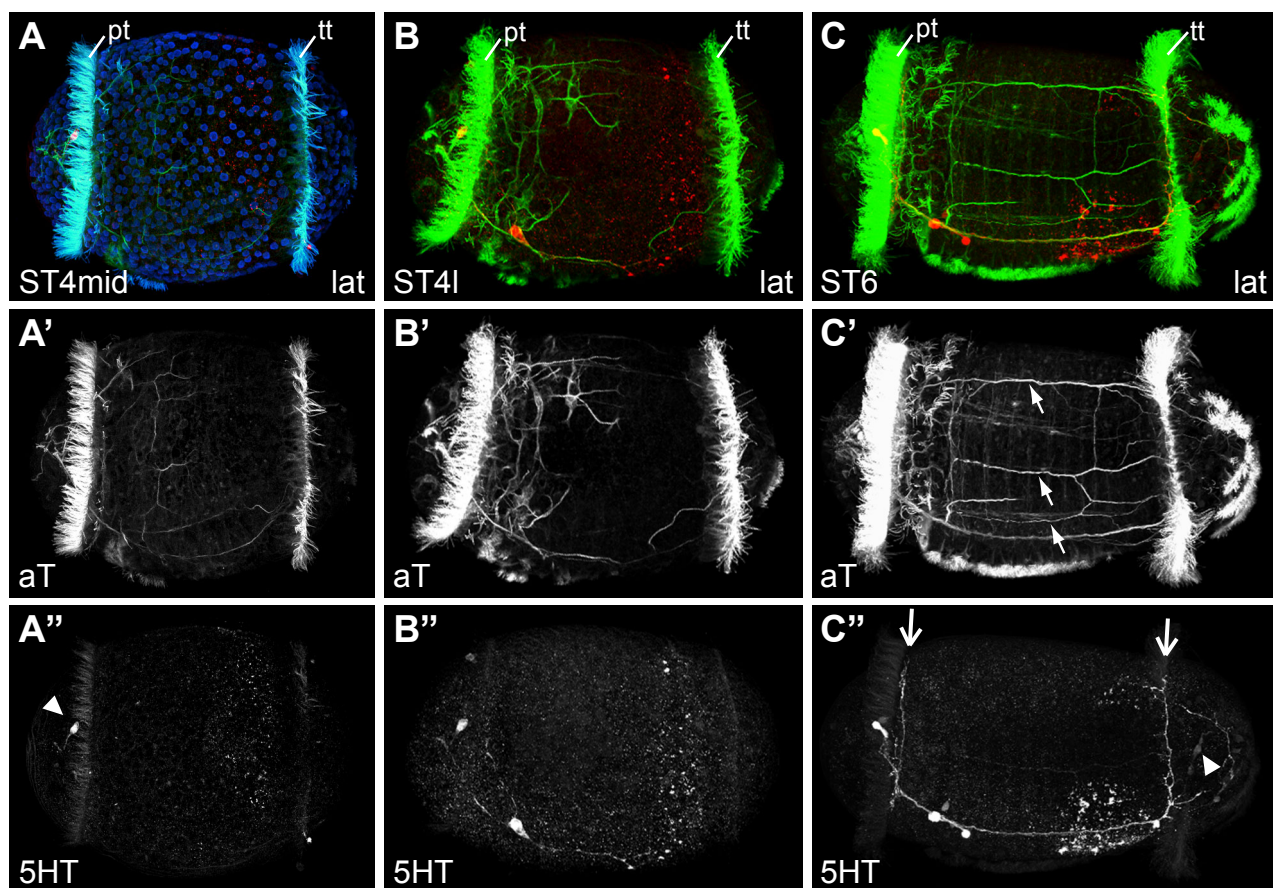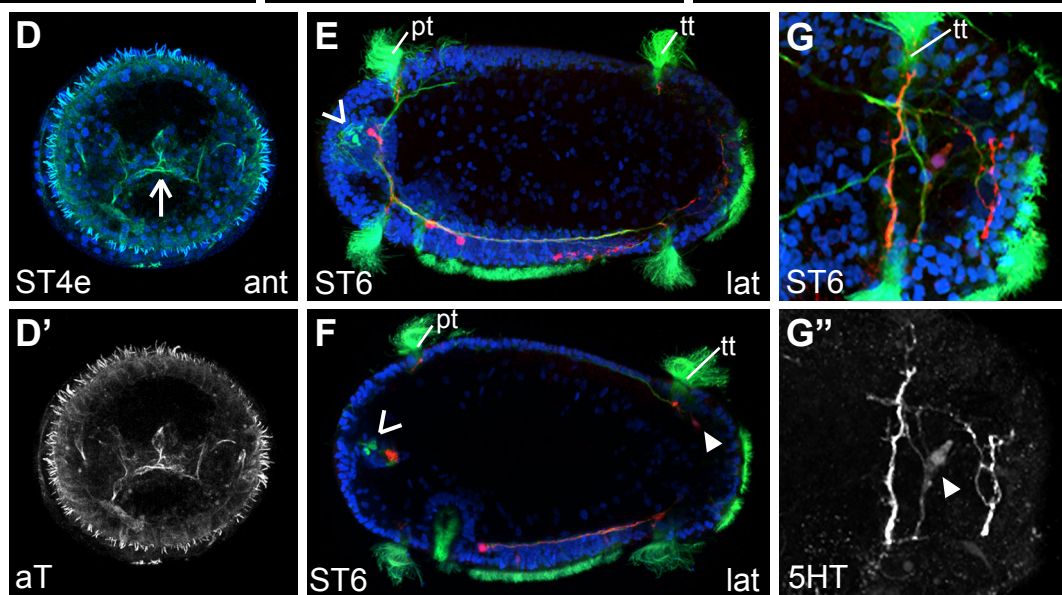

Supplement: Additional file 1: — aTUB-LIR and 5HT-LIR in early-stage C. teleta larvae (stages 4 – 6). Images are z-stack confocal images of larvae labeled with anti-acetylated-α-tubulin (green), anti-serotonin (red) and anti-histone (blue). Panels labeled with an apostrophe (e.g., A') are single-channel images of either aTUB-LIR (’) or 5HT-LIR (”) from the merged image without an apostrophe (e.g., A). All panels are to the same scale unless otherwise noted. G is a cropped, 2.7× magnified view of the pygidium. The position of the prototroch and telotroch is indicated in A, B, C, E and F, and the position of the telotroch is indicated in G. The closed arrowhead in A” points to the left soma with 5HT-LIR in the brain. The closed arrows in C’ point to the left longitudinal neurites in the dorsal-lateral and ventral-lateral trunk. The open arrows in C” point to neurites with 5HT-LIR that run along the prototroch and telotroch. The closed arrowhead in C” and G” points to S-PC. The open arrow in D points to the first neurites with aTUB-LIR. The open arrowhead in E and F marks the scac+. The closed arrowhead in F points to a posterior cell body with 5HT-LIR. Stage is indicated in the lower-left corner, and view is indicated in the lower-right corner of the merged panels. All lateral views are of the left side. Anterior is to the left in all lateral views, and ventral is down in all anterior views. ant, anterior; lat, lateral; pt, prototroch; tt, telotroch. [file 12983_2015_108_MOESM1_ESM.pdf]

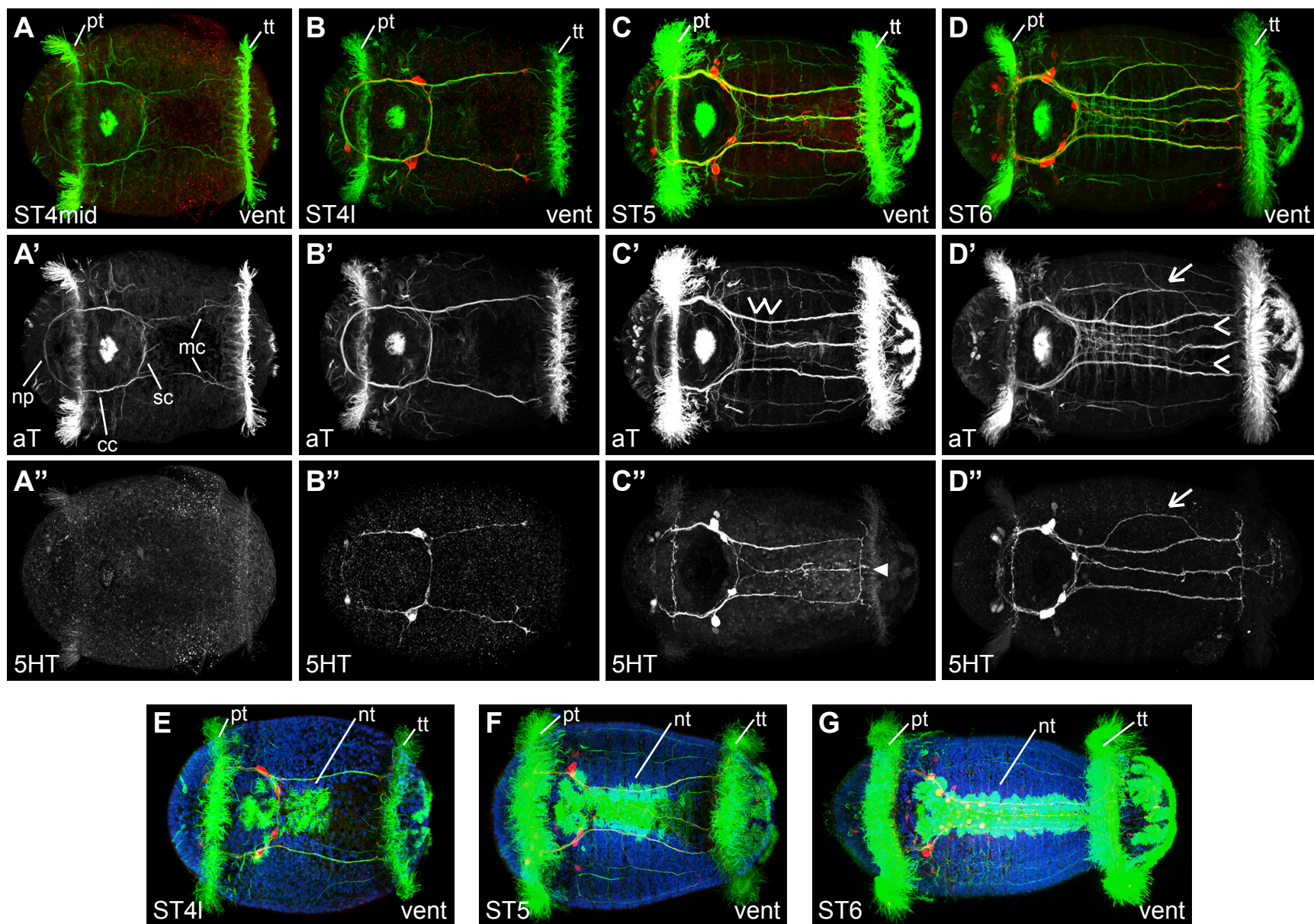

Supplement: Additional file 2: — Progression of the ventral nerve cord from stage 4 – 6 (aTUB-LIR and 5HT-LIR). Images are z-stack confocal images of larvae labeled with anti-acetylated-α-tubulin (green), anti-serotonin (red) and nuclear stain (blue). Panels labeled with an apostrophe (e.g., A') are single-channel images of either aTUB-LIR (’) or 5HT-LIR (”) from the merged image without an apostrophe (e.g., A). The position of the prototroch and telotroch is indicated in A – G. The brain neuropil (np), circumesophageal connectives (cc), subesophageal commissure (sc) and main connectives (mc) of the ventral nerve cord are indicated in A’. The open arrowheads in C’ point to forming commissures, and the closed arrowhead in C” indicates the position of the ventromedian connective. The open arrows in D’ and D” point to an aberrant neurite, while the open arrowheads in D’ point to the paramedian connectives. Panels E – G show the lengthening and narrowing of the neurotroch (nt); the prototroch (pt) and telotroch (tt) are also indicated. All are ventral views with anterior to the left. Stage is indicated in the lower-left corner. cc, circumesophageal connectives; mc, main connectives; np, neuropil; pt, prototroch; sc; subesophageal commissure; tt, telotroch; vent, ventral. [file 12983_2015_108_MOESM2_ESM.pdf]

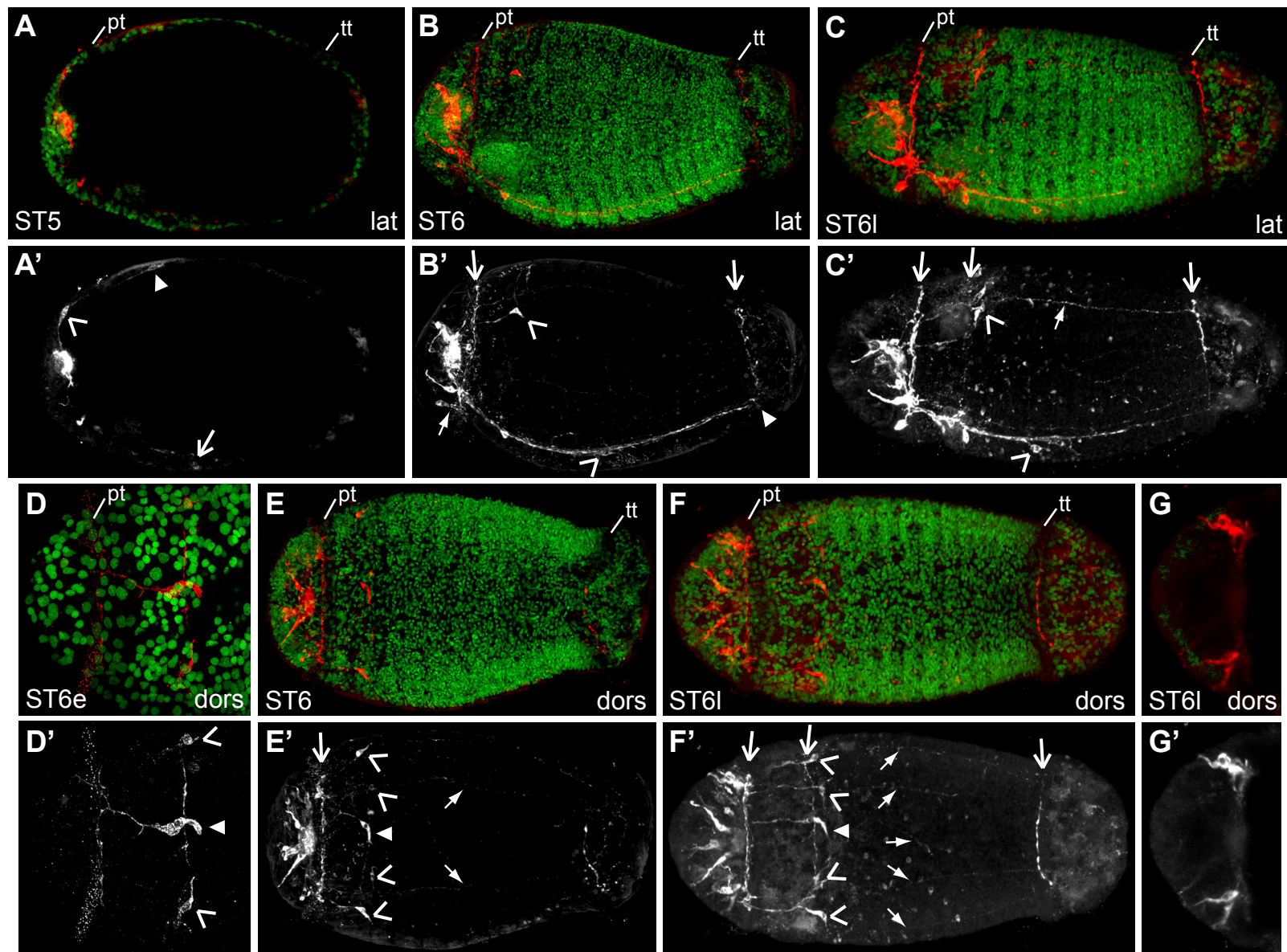

Supplement: Additional file 3: — FMRF-LIR in early-stage C. teleta larvae (stages 4 – 6). Images are z-stack confocal images of larvae labeled with anti-FMRF (red) and Hoechst nuclear stain (green). Panels labeled with an apostrophe (e.g., A') are single-channel images of FMRF-LIR from the merged image without an apostrophe (e.g., A). All panels are to the same scale unless otherwise noted. Panel D is a cropped, 2.2× magnified image of the head. The position of the prototroch and telotroch is indicated in A – C, E and F, and the position of the prototroch is indicated in D. The open arrowhead in A’ points to a dorsal-medial cell with FMRF-LIR. F-DMC is marked by a closed arrowhead in A’, D’, E’, F’. The first visible cells with FMRF-LIR in the ventral nerve cord are indicated with an open arrow in A’ and the lower open arrowhead in B’ and C’. In B’, a closed arrow points to a ventral-anterior flask-shaped cell with FMRF-LIR in the head, and a closed arrowhead marks a posterior cell near the ventral telotroch. Dorsal-lateral cells with FMRF-LIR are marked in B’ with the upper open arrowhead and in D’, E’, F’ with open arrowheads. Neurites that form a circumferential ring underlying the prototroch (B’, C’, E’, F’), in the mid-anterior dorsal trunk (C’, F’) and underlying the telotroch (B’, C’, F’) are labeled by the anterior, middle and posterior upper open arrows, respectively. Longitudinal dorsal trunk neurites are marked with closed arrows in C’, E’, F’. Stage is indicated in the lower-left corner, and view is indicated in the lower-right corner. All lateral views are of the left side. Anterior is to the left in all views. dors, dorsal; lat, lateral; pt, prototroch; tt, telotroch. [file 12983_2015_108_MOESM3_ESM.pdf]

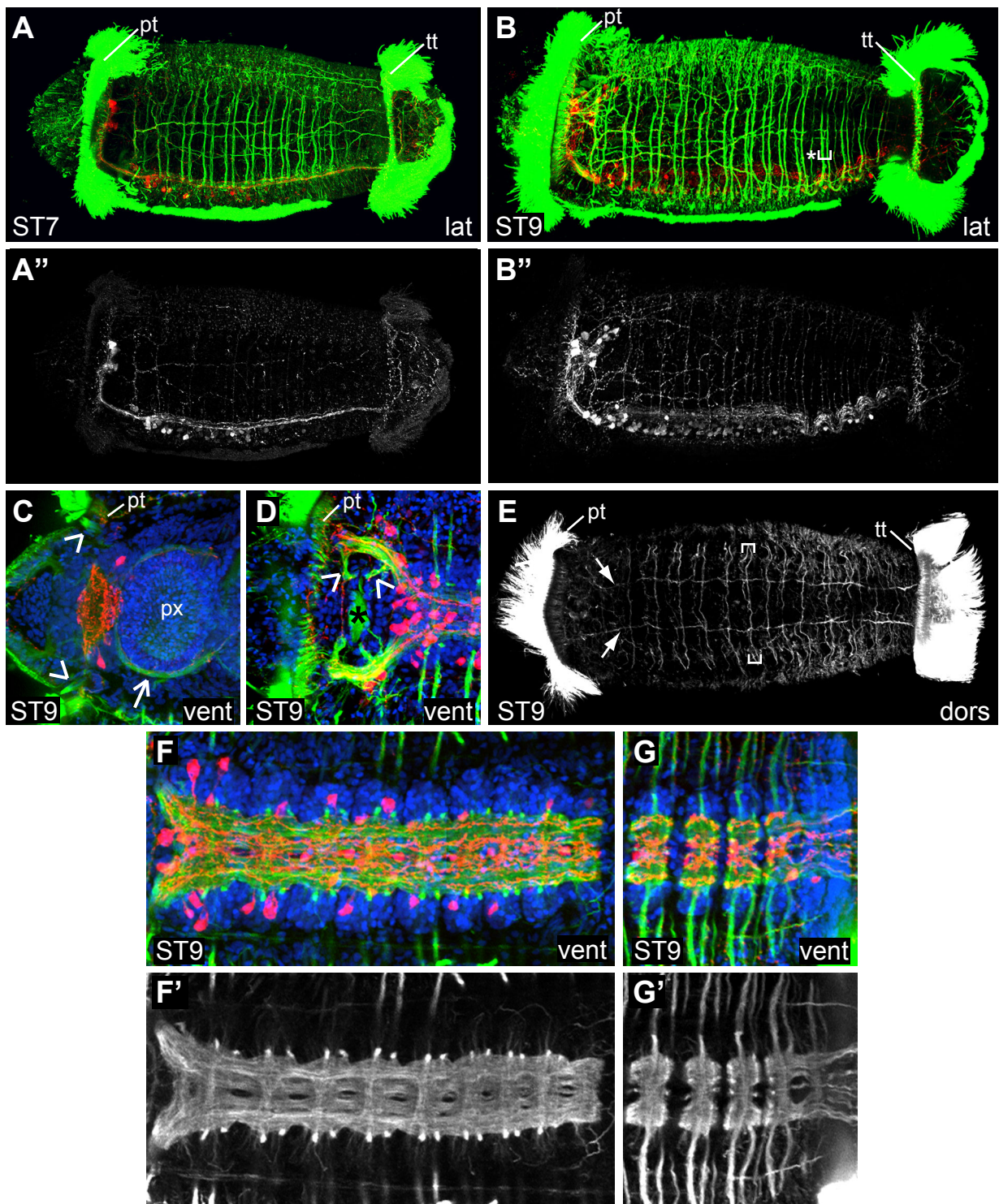

Supplement: Additional file 4: — aTUB-LIR and 5HT-LIR in late-stage C. teleta larvae (stages 7 – 9). Images are z-stack confocal images of larvae labeled with 1) anti-acetylated-α-tubulin (green) and anti-serotonin (red; A, B), 2) anti-α-acetylated-tubulin (green), anti-serotonin (red) and TO-PRO-3 nuclear stain (blue; C, D, F, G), or 3) anti-acetylated-α-tubulin (E). Panels labeled with an apostrophe (e.g., A') are single-channel images of either aTUB-LIR (’) or 5HT-LIR (”) from the merged image without an apostrophe (e.g., A). All panels are to the same scale unless otherwise noted. C and D are cropped, 2.27× magnified images of the head at stage 9. F is a cropped, 2× magnified image of the thoracic ganglia (1 – 9), and G is a cropped, 2× magnified view of the abdominal ganglia (10 – 13). The position of the prototroch and telotroch is indicated in A, B and E, and the position of the prototroch is indicated in C and D. In B, the bracket marks the paired main peripheral nerves, and the asterisk marks the minor peripheral nerve in segment 11. In C, the dorsal pad of the pharynx is labeled with a “px”, the open arrowheads point to the nuchal organs, and the open arrow points to the right anterior enteric nerve (aEN). The asterisk in D marks the mouth opening, and the open arrowheads point to a left pair of nerves that extend from the circumesophageal connectives towards the pharynx. In E, the brackets mark a pair of main peripheral nerves. The closed arrows in E point to dorsal-lateral longitudinal neurites with aTUB-LIR. Stage is indicated in the lower-left corner, and view is indicated in the lower-right corner. All lateral views are of the left side. Anterior is to the left in all views. lat, lateral; pt, prototroch; px, pharynx; tt, telotroch; vent, ventral. [file 12983_2015_108_MOESM4_ESM.pdf]

SFig 5

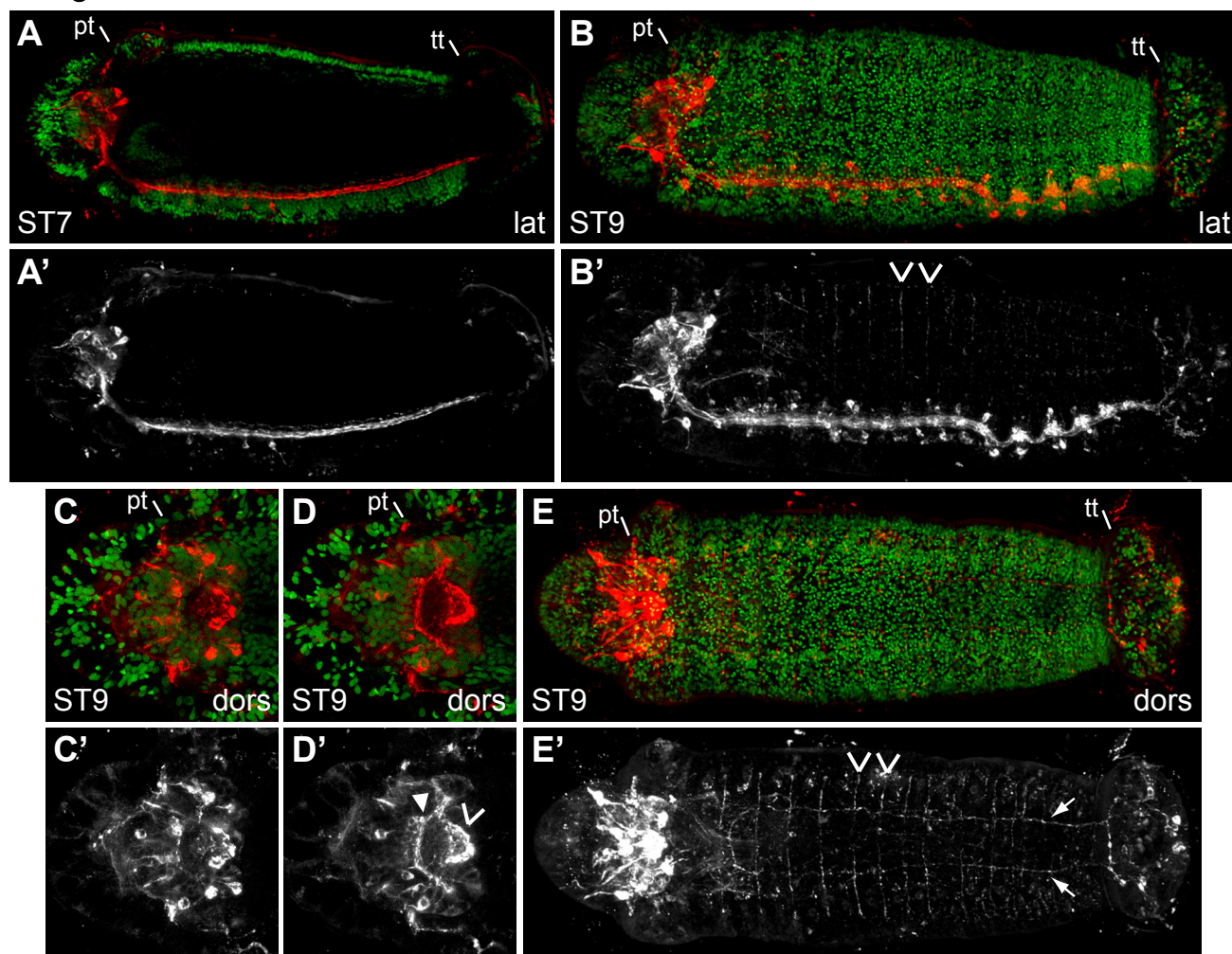

Supplement: Additional file 5: — FMRF-LIR in late-stage C. teleta larvae (stages 7 – 9). Images are z-stack confocal images of larvae labeled with anti-FMRF (red) and Hoechst nuclear stain (green). Panels labeled with an apostrophe (e.g., A') are the single-channel images of FMRF-LIR from the merged image without an apostrophe (e.g., A). All panels are to the same scale unless otherwise noted. C and D are cropped, 1.3× magnified images of the brain. The position of the prototroch and telotroch is indicated in A, B and E, and the position of the prototroch is indicated in C and D. Open arrowheads in B’ and E’ point to two segmentally-iterated neurites that are positioned beneath the ectoderm. In D’, the closed arrowhead points to a ventral-anterior tract with FMRF-LIR in the brain neuropil, and an open arrowhead points to a dorsal-posterior tract. Closed arrows in E’ point to dorsal-lateral longitudinal neurites that are positioned beneath the ectoderm. Stage is indicated in the lower-left corner, and view is indicated in the lower-right corner. All lateral views are of the left side. Anterior is to the left in all views. lat, lateral; dors, dorsal; pt, prototroch; tt, telotroch. [file 12983_2015_108_MOESM5_ESM.pdf]

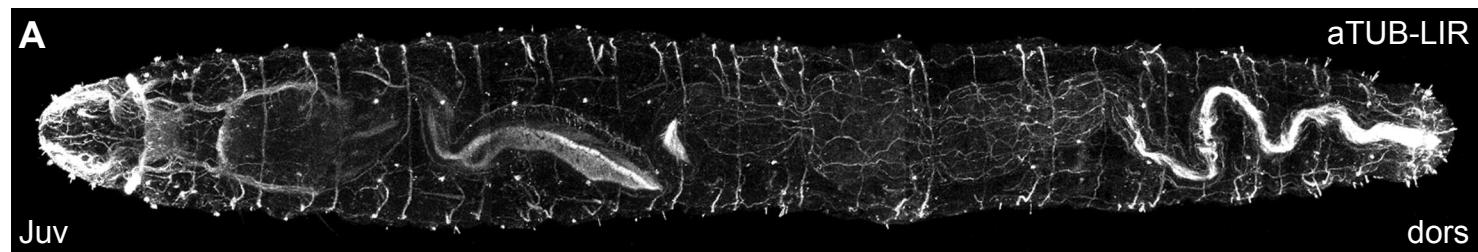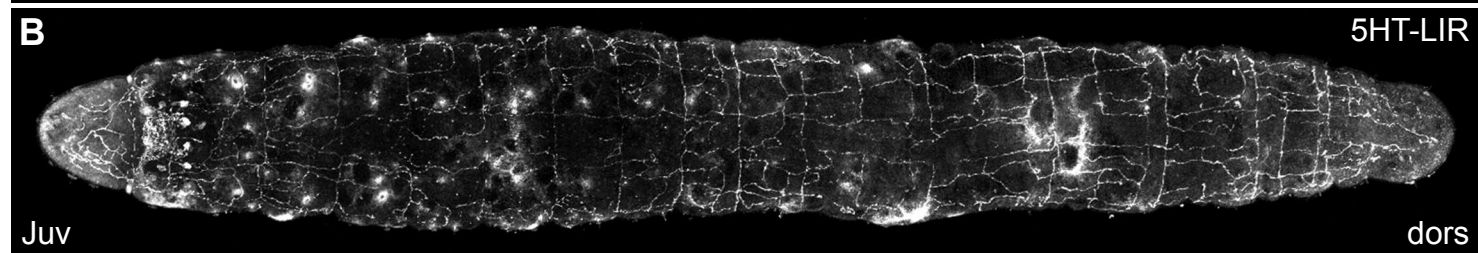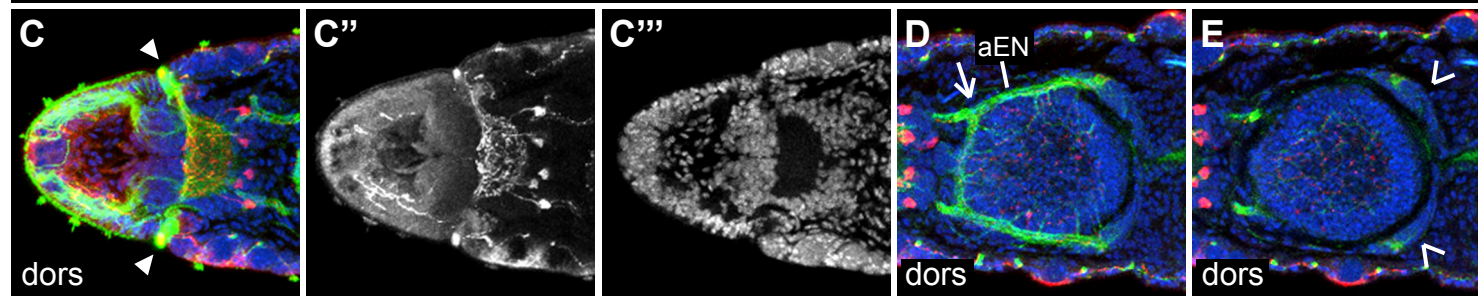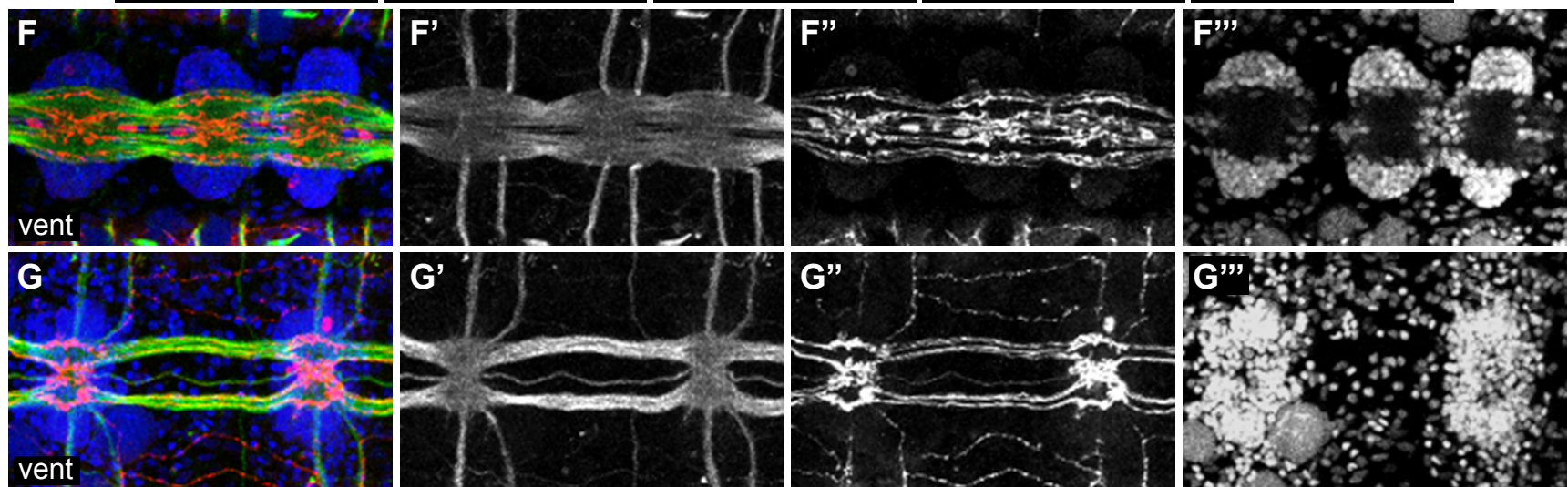

Supplement: Additional file 6: — aTUB-LIR and 5HT-LIR in 7-day old C. teleta juveniles. Images are z-stack confocal images of 7-day old juveniles labeled with anti-acetylated-α-tubulin (green), anti-serotonin (red) and TO-PRO-3 nuclear stain (blue). A is a single-channel image showing anti-acetylated-α-tubulin and B is a single-channel image of anti-serotonin. Panels labeled with an apostrophe (e.g., A') are single-channel images of either aTUB-LIR (’), 5HT-LIR (”) or TO-PRO-3 (”’) from the same z-stack as the animal in the panel without an apostrophe except for F’, which includes additional superficial focal planes to show the peripheral nerves. All panels are to the same scale unless otherwise noted. C is a cropped, 2.14× magnified image of the head; D and E are cropped, 2.14× magnified images of the pharyngeal dorsal pad; F is a cropped, 3.75× magnified images of thoracic ganglia 5 – 7; G is a cropped, 3.75× magnified image of abdominal ganglia 11 and 12. The closed arrowheads in C mark the nuchal organs. The open arrow in D points to the first branchpoint of the anterior enteric nerve (aEN). The open arrowheads in E indicate the stomatogastric ganglia. In A and B, stage is indicated in the lower-left corner, and view is indicated in the lower-right corner. In C – G, view is indicated in the lower left corner. Anterior is to the left in all views. dors, dorsal; vent, ventral. [file 12983_2015_108_MOESM6_ESM.pdf]

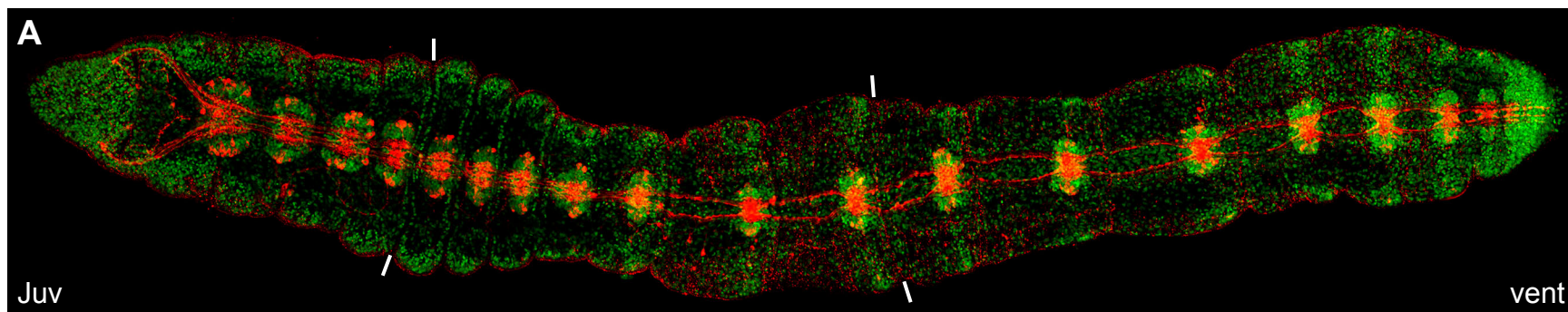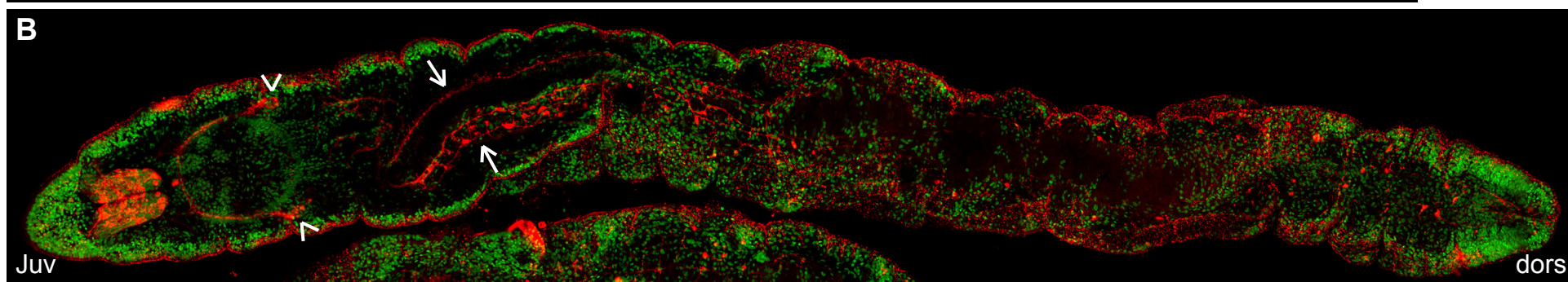

Supplement: Additional file 7: — FMRF-LIR in 7-day old C. teleta juveniles. Images are z-stack confocal images of 7-day old juveniles labeled with anti-FMRF (red) and Hoechst nuclear stain (green). In A, the posterior boundaries of segments 4 and 11 are indicated with lines. In B, open arrowheads point to clusters of neurons with FMRF-LIR near the pharynx, and open arrows point to the esophagus. Stage is indicated in the lower-left corner, and view is indicated in the lower-right corner. Anterior is to the left in all views. dors, dorsal; vent, ventral. [file 12983_2015_108_MOESM7_ESM.pdf]
